# French Phonological Component Analysis and aphasia recovery: A bilingual perspective on behavioral and structural data

**DOI:** 10.3389/fnhum.2022.752121

**Published:** 2022-09-22

**Authors:** Michèle Masson-Trottier, Tanya Dash, Pierre Berroir, Ana Inés Ansaldo

**Affiliations:** ^1^Laboratoire de Plasticité Cérébrale, Communication et Vieillissement, Centre de Recherche de l’Institut de Gériatrie de Montréal, Montréal, QC, Canada; ^2^École d’Orthophonie et d’Audiologie, Faculté de Médecine, Université de Montréal, Montréal, QC, Canada

**Keywords:** French Phonological Component Analysis, post-stroke aphasia, bilingual aphasia, cognitive control, recovery

## Abstract

Studies show bilingualism entails an advantage in cognitive control tasks. There is evidence of a bilingual advantage in the context of aphasia, resulting in better cognitive outcomes and recovery in bilingual persons with aphasia compared to monolingual peers. This bilingual advantage also results in structural changes in the right hemisphere gray matter. Very few studies have examined the so-called bilingual advantage by reference to specific anomia therapy efficacy. This study aims to compare the effect of French-Phonological Component Analysis (Fr-PCA) in monolinguals and bilingual persons with aphasia, both at the linguistic and cognitive control level, and to examine the structural impact of left hemisphere lesion location and right hemisphere structural data. Eight participants with chronic aphasia received Fr-PCA for a total of 15 h over 5 weeks. The results showed improved accuracy for treated words and generalization to untreated items and discourse in both groups, and improved Flanker task performance for some participants. Bilingual participants improved more than monolinguals for picture-naming tasks and narrative discourse. Damage to the left postcentral gyrus and the middle frontal gyrus was associated with less therapy-induced improvement. Additionally, left hemisphere damage to the inferior parietal gyrus and postcentral gyrus was associated with reduced cognitive control pre-therapy. Undamaged right hemisphere cortical thicknesses were significantly different between groups; the inferior frontal gyrus and the middle frontal gyrus were greater for the bilingual participants and correlated with cognitive control skills. These results suggest a bilingual advantage in anomia recovery following Fr-PCA, potentially resulting from enhanced cognitive control abilities that could be supported by right hemisphere neural reserve.

## Introduction

Aphasia is an acquired language disorder following brain injury occurring in over 1/3 of hospitalized stroke patients; an estimated 165,000–380,000 Canadians are affected with chronic aphasia (Simmons-Mackie, 2017). Aphasia can lead to various degrees of difficulty in speaking, understanding, reading, or writing. Among the plethora of symptoms, the most common, persistent, and debilitating symptom is anomia – a difficulty in finding words. Speech and language therapy (SLT) effectively improves anomia [see Brady et al. (2016) for a meta-analysis], but there is little consensus on which anomia therapies are most effective and efficient.

Over half the world’s population is considered bilingual (Grosjean, 2015), and in Canada, 19.4% of the population speaks two or more languages at home (Statistic Canada, 2017). The increasing bilingual population also leads to a higher incidence of bilingual persons with aphasia (Ansaldo and Saidi, 2014). Research shows that knowing more than one language entails a cognitive advantage at the executive function level (Bialystok et al., 2004; Abutalebi and Green, 2007; Ansaldo et al., 2015; Zhou and Krott, 2016; Dash et al., 2019; DeLuca et al., 2019), which induces neuroplasticity and structural adaptations (Abutalebi and Green, 2016). Studies on neurologically intact bilingual adults report better cognitive control^1^ than monolinguals, referred to as the bilingual advantage (Bialystok et al., 2004; Bialystok, 2009; Costa et al., 2009; Ansaldo et al., 2015; Berroir et al., 2017). In addition, more studies are pointing toward anatomical differences between bilingual and monolingual adults in cortical and subcortical grey matter structures (Mechelli et al., 2004; Li et al., 2014; Abutalebi and Green, 2016; Olulade et al., 2016; Marin-Marin et al., 2022). According to these results, bilingualism is comparable to other life-long experiences that significantly alter the structural makeup of the brain when learning and maintaining a new skill (Marin-Marin et al., 2022). This advantage is also present in gray matter structures in both left and right hemispheres (Li et al., 2014; Abutalebi and Green, 2016). Studies using structural magnetic resonance imaging (MRI) found a difference in the cortical morphology of the bilateral anterior cingulate cortex, the left anterior temporal lobe, bilateral cerebellum, the left caudate nucleus, the left Heschl’s gyrus, the left inferior frontal gyrus (IFG) and the left inferior parietal lobe when comparing bilingual and monolingual participants (Li et al., 2014). Olulade et al. (2016) found greater gray matter volume for the IFG and middle frontal gyrus (MFG) in the right hemisphere (among others) in neurologically intact bilinguals when compared to monolinguals. The increased structural density in critical areas related to language processing and cognitive control, along with the correlations between structural density and behavioral performances such as naming and grammatical processing in a second language (Abutalebi et al., 2014; Pliatsikas et al., 2014), and other measures of bilingualism such as the age of acquisition and proficiency (Mechelli et al., 2004), provide substantial evidence for the bilingual cognitive advantage hypothesis.

Over the years, many studies have discussed predictors of aphasia recovery (Watila and Balarabe, 2015; Doogan et al., 2018) – aphasia-related, such as initial severity and aphasia type (Lazar et al., 2010), lesion-related (Sims et al., 2016; Benghanem et al., 2019; Sul et al., 2019), and, more recently, bilingualism-related (Lahiri et al., 2020). Another aphasia-related factor recently discussed is the level of cognitive control skills in persons with aphasia (PWA; Villard and Kiran, 2018; Simic et al., 2019). In a systematic review, Simic et al. (2019) found that PWA’s baseline executive control and linguistic skills appear to predict therapy success. Cognitive control is critical in showing consistent attention necessary to benefit from therapy (Villard and Kiran, 2018). Initial impairment severity has also been linked to the therapy outcome, where the more severe the aphasia, the less likely the PWA is expected to improve. Furthermore, studies looking at lesion size and location have reported that bigger lesions lead to more severe deficits (Watila and Balarabe, 2015), and lesions in the left IFG (Sims et al., 2016; Daria et al., 2019), the middle temporal gyrus (MTG; Sims et al., 2016; Daria et al., 2019), the superior temporal gyrus (STG; Daria et al., 2019) and angular gyrus (AnG)/supramarginal gyrus (SMG; Sims et al., 2016) lead to more severe aphasia. Interestingly, studies have also shown changes in grey matter structures of posterior dorsal stream language homologues in the right hemisphere (RH), resulting in better language production abilities in chronic aphasia (Xing et al., 2016). The role of RH is often discussed with respect to stroke-induced aphasia recovery. The recruitment of homotopic areas in the RH is one of the effective methods of post-stroke reorganization leading to recovery. It is commonly hypothesized that RH recruitment and overall lesion size in the left hemisphere may be related (Heiss et al., 1999; Anglade et al., 2014). Moreover, studies looking at treatment-induced brain plasticity in chronic aphasia provide additional evidence for RH recruitment to assist language recovery, revealing higher RH activity linked with treatment gains (Fridriksson et al., 2006, 2007; Meinzer et al., 2007; Kiran et al., 2015).

To this day, there remains little consensus in the literature, much less in the bilingual literature, on the predictive value of variables such as lesion size, lesion location, and role of the RH. Recent studies on bilingual persons with aphasia (bPWA) report a bilingual advantage in cognitive performances in stroke survivors (Dash and Kar, 2014; Alladi et al., 2016; Paplikar et al., 2018; Dekhtyar et al., 2020; Lahiri et al., 2020; Penaloza et al., 2020; Mooijman et al., 2021). Concerning aphasia symptom severity, bPWA have milder language deficits in their mother tongue (Paplikar et al., 2018; Ardila and Lahiri, 2020). These results suggest that bPWA show better cognitive control, less severe aphasia, and more recovery. However, the relationship between cognitive control, word retrieval, and bilingualism remains unclear based on the recent findings from Faroqi-Shah et al. (2018), which showed a lack of association between Stroop performance and both category fluency and picture-naming in PWA. The interplay between such bilingual advantage and the potential benefits of specific therapy approaches remains to be understood, especially knowing that cognitive control could be a significant predictor of recovery (Simic et al., 2019).

In impairment-based therapies targeting anomia, phonological therapies, such as Phonological Component Analysis (PCA; Leonard et al., 2008; Madden et al., 2017; Kristensson and Saldert, 2018; Marcotte et al., 2018), aim to facilitate lexical retrieval by activating the phonological representation of words (Goldrick and Rapp, 2002) and by assimilating the trained cueing strategies. Leonard et al. (2008) developed the PCA protocol as the phonologically oriented version of semantic feature analysis (Boyle and Coelho, 1995; Coelho et al., 2000); it includes a series of five cues based on phonological components of the target word. As per the original therapy protocol, the multiple consecutive phonological cues in PCA are cognitively demanding and require both flexibility and inhibition. Studies have linked therapy gains with PWA’s cognitive control associating it with their ability to benefit from the trained cues (Gilmore et al., 2019; Simic et al., 2019). Moreover, generalization following ortho-phonological cues has been related to inhibition – a subcomponent of cognitive control (Yeung and Law, 2010). Previous case studies with PCA have established the treatment efficacy in terms of acquisition, improvement as measured by treated items (Leonard et al., 2008; Bose, 2013; Kristensson and Saldert, 2018), within-level generalization, improvement as measured by untreated items (Leonard et al., 2008), and across-level generalization, improvement as measured by linguistic tasks other than the one targeted in treatment such as discourse (Kristensson and Saldert, 2018). For a detailed paper on the different levels of generalization, please see Webster et al. (2015).

Studies have been performed using phonological approaches to treat anomia in bilingual PWA and have reported good results, focusing on cross-linguistic therapy effects (Abutalebi et al., 2009; Croft et al., 2011). To this day, there has been little development in specific therapy-induced recovery studies in bPWA. In general, studies in bilingual aphasia recovery have been criticized for lack of methodological rigorousness (Faroqi-Shah et al., 2010; Simic et al., 2019), hence limiting the interpretations from these studies and generalizations possible. The only paper to this date investigating the impact of bilingualism on aphasia recovery found that bPWA improved more than their monolingual counterparts from the acute phase (3–7 days post-stroke) to the sub-acute phase (90–100 days post-stroke) (Lahiri et al., 2020). However, there is limited information on the potential bilingual advantage in aphasia recovery in the chronic phase. Moreover, considering the cognitive control advantage reported in bilinguals, and given the large proportion of bilinguals in the world population, it is important to improve the understanding of the relation between the cognitive control performance and naming therapy outcome in monolingual and bilingual PWA.

This study aims to investigate the effects of Phonological Component Analysis delivered in French (Fr-PCA) with French monolingual (mPWA) and French-English bPWA on their linguistic and cognitive profiles, in relation to their respective lesion locations, and cortical thickness in the undamaged hemisphere. The specific questions concerning the efficacy of Fr-PCA, the lesion location’s impact, and the RH’s role addressed in this paper and their corresponding hypotheses are listed below. For each research question, we are interested in looking at improvement or performance for all participants, and across groups (mPWA vs. bPWA) when possible, allowing us to evaluate the efficacy of Fr-PCA and the potential influence of bilingualism.

### Efficacy of French-Phonological Component Analysis

Does Fr-PCA lead to improvements in naming accuracy for treated items (*acquisition*), untreated items and other object naming tasks (*within-level generalization*), and other linguistic abilities and cognitive tasks (*across-level generalization and transfer*)? If so, do bPWA show more significant treatment effects (in *acquisition, within- and across-level generalization and transfer*) when compared to mPWA?

In line with previous research on the English version of PCA (Leonard et al., 2008; Bose, 2013; Kristensson and Saldert, 2018; Marcotte et al., 2018), it is hypothesized that Fr-PCA will improve naming abilities for treated (*acquisition*) and untreated words (untreated items and TDQ60; *within-level generalization*), as reflected by higher accuracy scores on picture-naming probes. It is also hypothesized that Fr-PCA will improve standardized language test scores, connected-speech main concept scores (*across-level generalization*), and cognitive control task performance (*transfer*). Moreover, in line with recent works and with the load of Fr-PCA on cognitive control, it is expected that improvement will be observed in both groups, with bilinguals showing better improvement levels (Gilmore et al., 2019; Lahiri et al., 2020).

### Impact of lesion location

Does lesion location play a role in pre-therapy cognitive-linguistic abilities and subsequent therapy outcomes?

In line with previous results, we hypothesize that more damage to regions of interest (ROIs) involved in language and cognitive control networks will lead to more severe linguistic and cognitive difficulties (Abutalebi et al., 2008; Luk et al., 2011; Daria et al., 2019). We thus expect significant correlations between pre-therapy cognitive-linguistic test outcomes and lesioned voxels in the ROIs. Furthermore, regarding therapy outcome, it is hypothesized that participants with lesions within the ROIs associated with lexical retrieval and phonological processing would improve less (Fridriksson, 2010).

### Role of the right hemisphere

Does RH cortical thickness vary across monolingual and bilingual PWA, and if so, does it relate to performance in cognitive and language tasks?

Given the role of RH in language processing in PWA (Lukic et al., 2017) and structural differences in bilinguals in key cognitive control regions (Felton et al., 2017), we wanted to assess the differences in RH regions between bPWA and mPWA. In line with work by Li et al. (2014), it is expected that cortical thickness in RH areas known for their role in cognitive control will show higher cortical thickness bPWA as compared to their monolingual peers and that such differences will correlate with scores on cognitive and linguistic tasks.

An exploratory research framework addresses the questions regarding the impact of lesion location (question 2) and the role of the RH (question 3).

## Materials and methods

### Design

This is a pre-, post-therapy study with multiple baselines and repeated measures before and after Fr-PCA (three baseline probes and one post-therapy probe) across groups. To answer the specific questions, we use a mixed design with case series analysis accompanied by group analysis. Specific methods are described in section “Data analysis plan.”

### Participants

Eight participants with aphasia took part in this study; four monolingual and four bilingual participants with aphasia (mPWA – bPWA), matched for age, education, and time post-aphasia onset (TPO). See Table 1 for demographic information. Groups did not differ significantly in lesion size or baseline linguistic profile [mean baseline picture-naming probes accuracy rate, object naming as measured with the TDQ60 (Macoir et al., 2017), verb naming as measured with the DVL38 (Hammelrath, 2005), oral comprehension measured with the MT86 subtest (Nespoulous et al., 1986), repetition measured with the MT86 subtest, verbal fluency measured with the MT86 subtest, and Cinderella storytelling main concepts score (Dalton and Richardson, 2015; Richardson and Dalton, 2016)]. Please see Supplementary Appendix A for detailed group comparison statistics.

**TABLE 1:** Demographic information, clinical data, and standardized neuropsychological background test scores of participants with aphasia.

ID	Gender Age (years)	Race and ethnicity	Education (years)	Handedness* (post morbid)	TPO (months)	Site of lesion	Size of lesion (mm3)	Type of aphasia and severity	Apraxia severity#	Baseline naming (%)	MoCA	CASP	Depression**
MA1	M ⋅ 60	White	12	Left	172	Left sylvian territory with no parietal lesion	143626	Anomic Mild	None	98%	27	36	Normal
MA2	F ⋅ 72	White	12	Right	47	Left sylvian territory with fronto-insular parietal, occipital lesion	39848	Broca’s Moderate to severe	Mild to moderate	16%	20	24.5	Severe
MA3	M ⋅ 73	White	6	Right	36	Left insula and parieto-temporal regions	1933	TMA Moderate to severe	Mild	39%	15	34	Normal
MA4	F ⋅ 70	White	15	Right	41	Left sylvian territory with frontoparietal lesion	74868	Global Severe	Moderate to severe	31%	n.a	34.5	Normal
BA1	M ⋅ 77	White	17	Right	11	Left temporal regions	10592	Anomic Moderate to severe	Mild	67%	20	34	Normal
BA2	F ⋅ 63	White	18	Right	11	Left MCA territory with insular, subinsular and frontal lesion	33455	Broca’s Mild to moderate	Mild to moderate	96%	26	33.5	Normal
BA3	M ⋅ 65	White	15	Both	57	Left sylvian territory	61021	Anomic Mild to moderate	None	89%	27	31	Normal
BA4	M ⋅ 48	White	15	Both	22	Left sylvian territory with fronto-insular parietal occipital lesion	15416	TMA Moderate	Mild	63%	24	35	Normal
Mean (*SD*) mPWA	68.75 (5.97)		11.25 (3.77)		74.00 (65.49)		65069 (60248)			46% (36%)	20.67 (6.03)	32.25 (5.24)	
Mean (*SD*) bPWA	63.25 (11.90)		16.25 (1.50)		25.25 (21.79)		30121 (22829)			79% (16%)	24.25 (3.10)	33.38 (1.70)	

Race and ethnicity as observed by the investigator. *Based on The Edinburgh inventory (Oldfield, 1971). TPO, time post-onset in months; Site of lesion as identified in radiology report; MCA, middle carotid artery; CVA, cerebrovascular accident; TMA, transcortical motor aphasia. *#*, Based on the Apraxia battery for adults 2 (ABA2, Dabul, 2000). Baseline naming is average accuracy in percentage at baseline assessments on the full 270 item baseline picture-naming probes described in section “Primary outcome measure: picture-naming probes.” MoCA, Montreal Cognitive Assessment, scored on 30, cut-off at 26; CASP, Cognitive Assessment scale for Stroke Patients, scored on 36, alert point at 35. **, Based on Geriatric Depression Scale (GDS; Yesavage et al., 1982).

**FIGURE 1:**
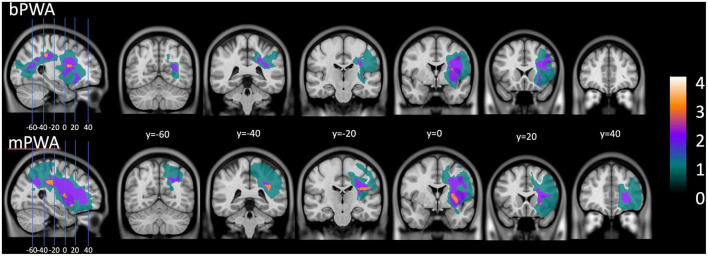
Lesion overlay plot: on the **(upper section)**, lesion size for all bilingual participants with aphasia. On the **(lower section)**, lesion size averaged for all monolingual participants with aphasia.

Mother tongue (L1) was French for all participants, and for bPWA, English was the second language (L2). All participants lived in the province of Quebec, a predominantly francophone province within the bilingual English-dominant country of Canada. Table 2 displays LEAP-Q language proficiency details. Figure 1 illustrates lesion overlay for both groups.

**TABLE 2:** Individual scores on language experience and proficiency questionnaire (LEAP-Q) for all participants.

ID	L2 AoA (years)	Exposure				L1						L2						BNT FR (/30)	BNT EN (/30)
		Pre		Post		Premorbid			Postmorbid			Premorbid			Postmorbid				
		L1	L2	L1	L2	E	OC	read	OE	OC	read	OE	OC	read	OE	OC	Read		
MA1		100		100		7	10	10	7	10	10							25	
MA2		100		100		8	8	8	2	10	6							2	
MA3		100		100		10	10	10	7	6	0							5	
MA4		100		100		10	10	10	3	6	5							6	
BA1	23	80	20	100	0	9	10	10	5	7	6	7	8	8	5	5	7	16	10
BA2	11	60	39	80	20	8	9	9	2	9	5	7	8	8	2	8	5	25	13
BA3	18	90	10	100	0	10	10	10	4	10	4	7	8	6	2	4	0	24	9
BA4	18	60	40	90	10	10	10	10	7	9	6	8	8	8	7	8	7	14	2

AoA, age of acquisition; OE, oral expression; OC, oral comprehension; read, reading.

Participants were recruited as part of a larger research project studying the efficacy of Fr-PCA through community-based aphasia associations and community advertising. They all presented with chronic aphasia following a single left hemisphere stroke (minimum of 6 months post-onset) and were MRI compatible. Participants did not have premorbid or concomitant neurological conditions (e.g., developmental language disorder, neurodegenerative disorder, TBI, etc.), uncorrected hearing, or vision loss. Throughout the study, they did not receive any other speech-language therapy or therapy of any sort.

The CRIUGM’s aging and neuroimagery research ethics committee (CMER RNQ 15-16-02) approved this project. All participants provided informed written consent to participate in the study.

### Assessments

Participants completed a comprehensive battery of language and cognitive tests before and after Fr-PCA therapy (see section “Language tasks” and Table 1 for battery tests). An SLP (MMT) administered the tests and split them into three 2.5-h assessment sessions to collect three picture-naming baselines and reduce fatigue. Still, some participants were unable to perform the Flanker task pre-therapy. Participants completed an adapted version of the Language Experience and Proficiency Questionnaire (Marian et al., 2007) with the SLP’s help and underwent a structural MRI (T1 image).

#### Primary outcome measure: Picture-naming probes

The baseline picture-naming probe comprises 270 pictures selected from the Bank of Standardized Stimuli (BOSS; Brodeur et al., 2011) and validated among healthy elderly French speakers (Masson-Trottier et al., 2016). It includes various categories such as fruits, vegetables, clothes, animals, body parts, furniture, and other objects. To be scored as a correct answer, participants had to name the picture correctly within 10 s (Evans et al., 2020). The baseline picture-naming probes were repeated in all three assessment sessions to generate two independent lists balanced for frequency, number of phonemes, and syllables (New et al., 2004). List 1 is composed of 20 items chosen with the participant to be treated in therapy, and List 2 is composed of 40 items not to be treated in therapy (i.e., to measure within-level generalization). Items selected for Lists 1 and 2 were named incorrectly on 2 or 3 baselines.

#### Secondary outcome measures

##### Language tasks

During the assessment sessions, several relevant language tasks were selected to be part of the comprehensive language assessment to characterize the type of aphasia in our participant sample and to measure the effect of Fr-PCA on within-level and across-level generalization (Webster et al., 2015). The tasks included in the comprehensive language assessment are the *Test de denomination de Québec-60* (TDQ60; Macoir et al., 2017) for object naming, the *Test de dénomination de verbes lexicaux en images-38* (DVL38; Webster et al., 2015) for verb naming, sub-tasks from the *Montreal-Toulouse* aphasia battery; oral comprehension, repetition, and verbal fluency with semantic criteria (Béland and Lecours, 1990), and a narrative discourse sample of the Cinderella story analyzed with the main concepts (MC) method (Dalton and Richardson, 2015; Richardson and Dalton, 2016).

The tasks used to measure within-level generalization are picture-naming accuracy variation on List 2 (untreated items) and performance variation on TDQ60, a francophone object-naming test. Tasks used to measure across-level generalization are the variation on DVL38, a verb naming test, on the sub-tasks in the MT86, and the variation on the narrative discourse main concept score.

##### Flanker task

Participants underwent the Eriksen’s Flanker task (Eriksen and Eriksen, 1974) presented via the E-Prime 2.0 software (Psychology Software Tools, Inc., Sharpsburg, PA). The Flanker task assesses participants’ ability to suppress interference from irrelevant non-verbal stimuli. Processing verbal stimuli is difficult for PWA, making the Flanker task suitable for this population. They were presented with an array of five arrows in the middle of the screen and asked to determine whether the central arrow pointed left or right. A target stimulus (i.e., central arrow), which points leftward or rightward, is surrounded by two flankers on each side. There are two flanker conditions: congruent (arrows that point in the same direction as the central arrow) and incongruent (arrows that point in the opposite direction from the central arrow). The Flanker task began with a short instruction phase followed by 20 practice trials and 240 experimental trials consisting of an equal number of congruent and incongruent trials. Each began with a fixation cross of 400 ms, followed by the target window. The stimuli appear at the center of the screen for 3000 ms. The next trial starts as soon as the participant responds or after 3000 ms (whichever comes first). For the Flanker test, the outcome measures are the Flanker effect (incongruent RT – congruent RT), indicating interference effect, response times (RT), and error rates for congruent and incongruent conditions.

#### MRI structural image acquisition

Images were acquired using a 3 T MRI Siemens Trio scanner, which was updated (Prisma Fit) with a standard 32-channel head coil during data collection. A high-resolution structural image was obtained using a 3D T1-weighted imaging sequence using an MP-RAGE (TFE) sequence (TR = 2300 ms; TE = 2.98 ms; 192 slices; matrix = 256 × 256 mm; voxel size = 1 × 1 × 1 mm; FOV = 256 mm).

##### MRI image preprocessing and anatomical measurements

The MRIs were acquired at the Functional Neuroimaging Unit in Montreal. For each participant, a lesion map was manually drawn, considered the golden standard for lesion mapping (Meinzer et al., 2013; Liew et al., 2021). FreeView visualization tool, a FreeSurfer (Fischl, 2012) feature, was used to perform the lesion mapping. On each axial slice, the border of the necrotic tissue was carefully delimited, and additional manual corrections were applied on coronal and sagittal planes to smooth the edge of the lesion volume and to remove enclaves of cerebrospinal fluid (CSF). Sometimes CSF can be mingled with necrotic tissue. The lesion volume was computed and stored for further analysis.

Since each MRI contained extensive lesions, we used the “Clinical toolbox” (Rorden et al., 2007) to replace the necrotic tissue with synthetic tissue, which was generated based on the gray and white matter distribution in the contralateral hemisphere. All MRI data were processed locally with FreeSurfer v6.0.0^2^, Linux Mint 17 Ubuntu 14.04_x86_64 (Fischl et al., 1999, 2004; Fischl, 2012). The Destrieux atlas was used to apply the sulco-gyral parcellation and to build a surface-based atlas that included 74 labels per hemisphere (Fischl, 2012). The results enabled the obtaining of the thickness of cortical ROIs, which was computed as the average of (1) the distance from each white surface vertex to their corresponding closest point on the pial surface (not necessarily at a pial vertex) and (2) the distance from the corresponding pial vertex to the closest point on the white surface (Fischl and Dale, 2000). Further, for the lesioned left hemisphere of each participant, the output volume was filtered with the original lesion map to remove the segmentation portions corresponding to synthetic tissue. Considering the objective of generating cortical thickness data for each participant’s ROIs in the native space, normalization was not performed.

### French-Phonological Component Analysis therapy

The PCA treatment protocol allows reproducibility of methodology. Fr-PCA was administered according to the standard protocol (Leonard et al., 2008) by an experienced SLP (MMT). The phonological components include: giving the *first sound* of the target (Question: “What sound does it start with?”), the *final sound* (”What sound does it end with?”), the *number of syllables* (”How many beats does the word have?”), providing a *first sound associate* (”What other word starts with the same sound?”) and a *rhyme* to the target (”What does this rhyme with?”) (Leonard et al., 2008).

Fr-PCA therapy started immediately after the assessment. Participants received Fr-PCA for the items on List 1 (see above) for 15 1-h face-to-face sessions, three sessions per week for 5 weeks. Participants were met either at their homes or the research center, whichever they preferred. The PWA was asked to generate the answers to each cue; if this was not possible, they could choose between three valid options to promote active participation (Hickin et al., 2002). All items on List 1 were repeated 1 to 4 times during the session according to the participant’s pace and tolerance. Fr-PCA was presented on a laptop and coded into a python program allowing automatic randomization of items from session to session and online scoring of naming performance for each item. The SLP controlled therapy display on the laptop, writing the cues on the therapy screen, rating the answers given by the PWA, and giving appropriate feedback during the therapy.

### Data analysis

For all standardized language tests (TDQ60, DVL38, repetition, verbal fluency, and oral comprehension), *z*-scores were calculated using the normative mean and standard deviation values for the participant’s age and education level (see Table 1). The following formula was used to calculate the *z*-scores where the mean and standard deviation are from the normative data available:


(1) z⁢s⁢c⁢o⁢r⁢e=(R⁢a⁢w⁢S⁢c⁢o⁢r⁢e-M⁢e⁢a⁢n)⁢/⁢S⁢t⁢a⁢n⁢d⁢a⁢r⁢d⁢D⁢e⁢v⁢i⁢a⁢t⁢i⁢o⁢n.

*Z*-scores allow comparing language abilities and improvements following Fr-PCA across age and education levels. As per standard clinical guidelines used in speech-language pathology, *z*-scores below – 1.5 SD were considered mild impairment, and *z*-scores below – 2 SD were considered severe impairment (Spaulding et al., 2012; Satoer et al., 2019). Raw scores can be found in the Supplementary material.

To describe narrative discourse, main concepts were identified in the narrative discourse for the Cinderella storytelling task and then scored for accuracy and completeness (Nicholas and Brookshire, 1995). A main concept that was accurate and complete received a score of 3, an accurate incomplete or inaccurate complete main concept was given 2, and an inaccurate incomplete was scored 1 (Richardson and Dalton, 2016). The main concept score is the sum of all main concepts.

#### Data analysis plan

Data analysis was completed on SPSS 26 (IBM Corp, 2019) using an α-value of 0.05. The data analysis plan is presented for each specific question. We used a mixed method case-series analysis to draw interpretation from converging evidence.

##### Efficacy of French-Phonological Component Analysis

To answer the question of therapy efficacy, a case series analysis was conducted to investigate the *acquisition* following Fr-PCA, and group analyses were additionally performed to answer the *within- and across-level generalization* and *transfer* questions.

###### Acquisition: Case series

Four analysis methods appropriate for single-subject research designs were used to investigate the effect of Fr-PCA on naming accuracy for treated items. The results of these methods were merged to indicate if there was *strong evidence* (if all methods were large or significant), *moderate evidence* (if two or three of the methods were large or significant), *weak evidence* (if only one of the methods was large or significant), or *no evidence* of a treatment effect.

First, visual inspection was performed to judge whether change across the treatment phase was large enough to be seen by the *naked eye* (Franklin et al., 1996). Changes in performance were determined to be substantial if: (1) the mean probe score was greater than the mean baseline score, (2) there was no overlap of probe scores with baseline phase scores, and (3) probe scores surpassed the extended baseline trend line. For the visual inspection, accuracy from baseline assessments (3), therapy sessions (15), and post-therapy assessment (1) are used.

Second, effect size (*d*) was calculated to determine clinical significance using the standardized difference approach (Allison et al., 1996; Neumann, 2018), where


(2) $$d=\left({\text{score}}_{p\,o\,s\,t}-{\text{mean}}_{b\,a\,s\,e\,l\,i\,n\,e}\right)\,/\,s\,t\,a\,n\,d\,a\,r\,d\,d\,e\,v\,i\,a\,t\,i\,o\,n_{b\,a\,s\,e\,l\,i\,n\,e}.$$

For the analysis of effect size, the data from both assessments are used (three baselines and post-therapy). Following the guidelines given by Beeson and Robey (2006), it is not possible to perform *d* calculation when standard deviation_*baseline*_ = 0. When this happened, we calculated a pooled standard deviation using the last three therapy sessions, where


standard deviationpooled=standard deviationbaseline+standard deviationtherapy 13−14−152.

An effect size equal to or greater than 4.0, 7.0, and 10.1 was considered a small, medium, or large magnitude of treatment effect (Beeson and Robey, 2006).

Third, the Tau *U* Test of Trend, a non-parametric method used to measure non-overlap between two phases (A and B), was used to compare baseline accuracy scores to intervention accuracy scores. This analysis was performed using the Tau-*U* Calculator (Vannest et al., 2016). For the calculation of Tau-*U*, accuracy from baseline assessments (3) and therapy sessions (15) are used.

Finally, individual participant naming accuracy (i.e., correct/incorrect) was analyzed by item, using the WEighted STatistics (WEST) method outlined by Howard et al. (2014). This method overcomes problems of autocorrelation inherent in repeated measures designs; a single weighted score representing repeated measurements of an item is obtained and analyzed using a one-sample *t*-test. More specifically, WEST-ROC and WEST-Trend were calculated to respectively verify that the rate of change (ROC) post-therapy is significantly greater than the expected null ROC at baseline while also accounting for existing trends in the data, and that existing linear trends in the data are accounted for, indicating significant improvement, over and above existing trends in the data (Howard et al., 2014). In this specific analysis, for WEST-ROC, item naming scores for the three baselines and post-therapy assessment were multiplied by factors of 2, –1, –4, and 3, whereas for WEST-Trend, three baselines and post-therapy assessment were multiplied by factors of –3, –1, 1, and 3. For the WEST procedure, 4 one-sample *t*-tests (one-tailed) were conducted per participant. Using the Holm–Bonferroni procedure, alpha was initially set at 0.05 and adjusted accordingly for all subsequent comparisons to 0.05/4 = 0.0125.

###### Within- and across-level generalization: Case series and group analysis

First, to assess within-level generalization, we calculated individual effect sizes and WEST statistics with the untreated items following the same method described above. Furthermore, to complete the within-level generalization and to assess across-level generalization, Wilcoxon signed-rank tests^3^ were performed with the pre-therapy and post-therapy language *z*-scores for all PWA to see if Fr-PCA significantly improved language performance.

To verify the potential impact of bilingualism on therapy within- and across-level generalization, a non-parametric independent sample Mann–Whitney *U* test was completed comparing pre- and post-therapy score differences (called variation score) between groups for standardized language tests z-scores(*variationscore* = z−score_post−therapy_−z−score_pre−therapy_).

###### Transfer: Group analysis

Finally, to investigate improvement *transfer* following Fr-PCA, a Wilcoxon signed-rank test was also performed for the pre- and post-therapy outcome measures of the Flanker task to examine therapy transfer. A non-parametric independent sample Mann–Whitney *U* test was applied to compare post-therapy Flanker outcome measures between bPWA and mPWA to verify the potential impact of bilingualism on cognitive control, given that 2 monolingual participants could not perform the Flanker task before therapy.

##### Impact of lesion location

Considering the variability in lesion location among participants with strokes, a subset of ROIs damaged in at least 2 mPWA and 2 bPWA (see section “MRI image preprocessing and anatomical measurements” MRI Image Preprocessing and Anatomical Measurements for procedure) was identified to perform the correlations described below. To account for potential differences in lesion location across groups (mPWAs and bPWAs), a Mann–Whitney *U* test was performed to compare the number of voxels within each identified ROI between groups.

To answer the question regarding the impact of lesion location on pre-therapy skills, a Spearman’s rank-order correlation was performed with the number of voxels in each ROI within the lesion mask and scores on language and cognitive performance tasks before therapy for all PWA. To assess the impact of the lesion location on therapy-induced improvements (within- and across generalization), a Spearman’s rank-order correlation was also performed between the number of voxels in each ROI within the lesion mask and variation scores on language performance tasks.

To interpret the Spearman’s correlation, *r*_s_ = 0.00–0.19 was considered a very weak correlation, *r*_s_ = 0.20–0.39, a weak correlation, *r*_s_ = 0.40–0.59, a moderate correlation, *r*_s_ = 0.60–0.79, a strong correlation, and *r*_s_ = 0.80–1.0, a very strong correlation (Schober et al., 2018).

##### Role of right hemisphere

Finally, to answer the question concerning the potential experience-induced RH compensatory neuroplasticity, a non-parametric independent sample Mann–Whitney *U* test was completed comparing cortical thickness between bPWA and mPWA (Felton et al., 2017). For ROIs with significantly different cortical thickness between groups, a Spearman’s rank correlation was performed between cortical thickness and language and cognitive control scores pre-therapy and corresponding variation scores.

## Results

The result section for this paper is outlined in three main sections. Firstly, the efficacy of Fr-PCA is investigated with both case series analysis and group comparisons in terms of acquisition (improvement on treated items – List 1), within-level generalization (improvement on untreated picture-naming accuracy; untreated items – List 2 and TDQ60), across-level generalization (improvement on other language abilities) and transfer (improvement on cognitive control task). Secondly, the impact of the lesion location on pre-therapy linguistic and cognitive performances is examined using correlations. Finally, the role of the RH is explored in relation to the status of bilingualism and pre-therapy linguistic and cognitive performances.

### Efficacy of French-Phonological Component Analysis

To determine the efficacy of Fr-PCA, a mixed method was employed. Case series analysis is used to examine the acquisition with treated items using visual inspection of the data, analysis of effect size, Tau-U Test of Trend, and WEST statistics.

Furthermore, therapy-induced generalization (within- and across-levels) is examined with case series analysis and group analysis. Within-level generalization (naming accuracy of untreated items) is also investigated with two case series methods: analysis of effect size and WEST statistics. Then, we investigate the effect of Fr-PCA by comparing pre-therapy scores on standardized language tests to post-therapy scores. Finally, we compare the improvements of mPWA and bPWA for a potential impact of bilingualism on therapy outcomes.

#### Acquisition

**FIGURE 2:**
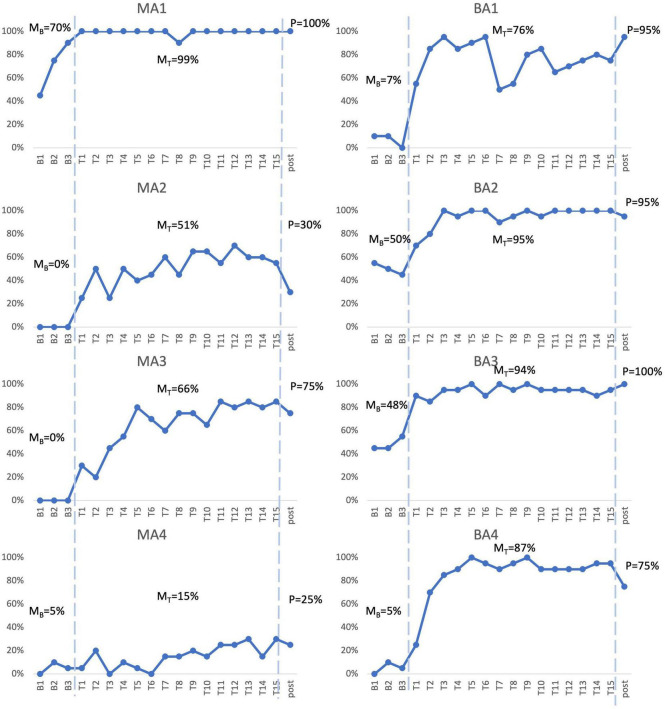
Picture-naming probe results. Repeated probe data for all participants. Graphs reflect percent accurate production for treated items at baseline assessments, each therapy session, and post-treatment assessment. M_B_ refers to mean accuracy of baseline assessments, M_T_ refers to mean accuracy of therapy sessions, P refers to accuracy at post-therapy assessment.

To examine acquisition following Fr-PCA, case-series analyses were performed. The summary of the case series analysis incorporating four analysis methods can be found in Table 3. Figure 2 shows each participant’s accuracy for items on list 1 used to measure acquisition. This figure is used for the visual inspection in the case study. Each participant will be discussed below.

**TABLE 3:** Case-study result summary: Evidence of acquisition and within-level generalization comparing baseline and treatment performance phases.

Participant		Visual inspection			Effect size	TAU u (*p*-value)	WEST-ROC						WEST-TREND					
						
		M_T_ > M_B_	No overlapping	Exceed baseline trendline			Mean	*SD*	*t*-value	df	CI 95	*p*-values one-tailed	Mean	*SD*	*t*-value	df	CI 95	*p*-values one-tailed
MA1	trtd	Yes	None	No	1.31	**0.99 (0.009)**	–0.45	2.01	–1.00	19	[–1.39–0.49]	0.165	1.80	1.64	4.90	19	[1.03–2.57]	0.000
	untrtd				1.03		–0.20	1.51	–0.84	39	[–0.68–0.28]	0.203	0.80	1.47	3.44	39	[0.33–1.27]	0.001
MA2	trtd	Yes	None	Yes	11.17***	**1.00 (0.008)**	**0.85**	**1.46**	**2.60**	**19**	**[0.17**–**1.53]**	**0.009**	**0.85**	**1.46**	**2.60**	**19**	**[0.17**–**1.53]**	**0.009**
	untrtd				26.06***		**1.05**	**1.45**	**4.58**	**39**	**[0.59**–**1.51]**	**0.000**	**1.05**	**1.45**	**4.58**	**39**	**[0.59**–**1.51]**	**0.000**
MA3	trtd	Yes	None	Yes	27.92***	**1.00 (0.008)**	**2.25**	**1.33**	**7.55**	**19**	**[1.63**–**2.87]**	**0.000**	**2.25**	**1.33**	**7.55**	**19**	**[1.63**–**2.87]**	**0.000**
	untrtd				18.61***		**0.75**	**1.32**	**3.61**	**39**	**[0.33**–**1.17]**	**0.000**	**0.75**	**1.32**	**3.61**	**39**	**[0.33**–**1.17]**	**0.000**
MA4	trtd	Yes	Yes	No	4.00*	0.75 (0.058)	0.45	1.88	1.07	19	[–0.43–1.33]	0.149	0.70	1.38	2.27	19	[0.05–1.35]	0.018
	untrtd				5.20*		0.23	0.92	1.55	39	[–0.07–0.52]	0.065	0.23	0.92	1.55	39	[–0.07–0.52]	0.065
BA1	trtd	Yes	None	Yes	15.30***	**1.00 (0.008)**	**2.65**	**0.75**	**15.90**	**19**	**[2.30**–**3.00]**	**0.000**	**2.15**	**1.90**	**5.06**	**19**	**[1.26**–**3.04]**	**0.000**
	untrtd				3.51		0.03	1.99	0.08	39	[–0.61–0.66]	0.469	0.90	1.58	3.60	39	[0.39–1.41]	0.000
BA2	trtd	Yes	None	Yes	9.00 **	**1.00 (0.008)**	**1.65**	**2.28**	**3.24**	**19**	**[0.58**–**2.72]**	**0.002**	**1.15**	**1.57**	**3.29**	**19**	**[0.42**–**1.88]**	**0.002**
	untrtd				5.44 *		0.25	2.15	0.74	39	[–0.44–0.94]	0.233	0.63	1.53	2.58	39	[0.14–1.11]	0.007
BA3	trtd	Yes	None	Yes	8.95 **	**1.00 (0.008)**	2.15	2.46	3.92	19	[1.00–3.30]	0.000	0.40	0.99	1.80	19	[–0.07–0.87]	0.044
	untrtd				6.05 *		**1.28**	**2.45**	**3.29**	**39**	**[0.49**–**2.06]**	**0.001**	**0.78**	**1.39**	**3.54**	**39**	**[0.33**–**1.22]**	**0.001**
BA4	trtd	Yes	None	Yes	14.00***	**1.00 (0.008)**	**1.95**	**1.61**	**5.43**	**19**	**[1.20**–**2.70]**	**0.000**	**2.20**	**1.32**	**7.44**	**19**	**[1.58**–**2.82]**	**0.000**
	untrtd				6.41 *		**1.05**	**2.10**	**3.16**	**39**	**[0.38**–**1.72]**	**0.002**	**1.05**	**1.77**	**3.76**	**39**	**[0.48**–**1.62]**	**0.000**

M_B_, refers to mean accuracy of baseline assessments; M_T_, refers to mean accuracy of therapy sessions. No overlap: Refers to whether treatment scores exceeded the baseline. Effect size that was equal to or greater than 4.0, 7.0, or 10.1 was considered a small, represented by *, medium, represented by **, or large magnitude of treatment effect, represented by ***, respectively, for lexical retrieval studies, as per Beeson and Robey (2006). Tau U: The number in parentheses is the two-sided *p*-value. In bold are the elements that provide evidence pour acquisition or within-level generalization.

MA1, the mPWA with the mildest aphasia (mild aphasia, no apraxia), demonstrated weak acquisition following Fr-PCA. Although MA1 showed a higher mean accuracy rate during therapy phase (99%) than baseline phase (70%) and no overlapping points between the phases, visual inspection revealed that points in therapy phase did not exceed baseline trendline, as he showed a noticeable improvement between baseline sessions in terms of accuracy. Indeed, after improving at each baseline assessment, MA1 reached ceiling effect at the first therapy session. This suggests that therapy performances do not surpass the improvement already initiated during baseline assessments. Even if WEST-Trend is significant, it is recommended that the effect of treatment be considered significant when both the WEST-Trend and WEST-ROC analyses yield significant results (Howard et al., 2014). Thus, given the non-significant result of WEST-ROC, this analysis is considered inconclusive, as is his effect size (ES = 1.31; non-significant). Finally, MA1 showed a significant Tau-*U* (Tau *U* = 1.00; *p* = 0.008), the only analysis method supporting acquisition for MA1. Although MA1 showed a limited gain in accuracy, Fr-PCA produced improvement in response time and pronunciation level, not reflected with the main outcome variable in this study.

MA2, who had moderate to severe aphasia and mild to moderate apraxia, showed strong evidence of acquisition following Fr-PCA. She demonstrated a greater mean accuracy rate during the therapy phase (51%) than during the baseline phase (0%), she did not show overlap in performance between therapy and baseline phases, and therapy improvements exceeded baseline trendline. When looking at the effect size, MA2 showed a large effect size for acquisition following Fr-PCA (ES = 11.17) and a significant Tau-*U* result (Tau *U* = 1.00; *p* = 0.008). Finally, both WEST-ROC and WEST-Trend yielded significant results. All four methods support MA2’s acquisition following Fr-PCA.

MA3, who presented moderate to severe aphasia and mild apraxia, showed strong evidence of acquisition following Fr-PCA. There was greater mean accuracy rate during the therapy phase [compared to baseline phase (0%)], with no overlap in performance between therapy and baseline phases. Therapy improvements exceeded baseline trendline. When looking at the effect size, MA3 showed a large effect size for acquisition following Fr-PCA (ES = 27.91) and a significant Tau-*U* result (Tau *U* = 1.00; *p* = 0.008). Finally, both WEST-ROC and WEST-Trend yielded significant results. All four methods support MA3’s acquisition following Fr-PCA.

MA4, who presented the most severe aphasia and moderate to severe apraxia, demonstrated no evidence of acquisition following Fr-PCA. Although MA4 showed a higher mean accuracy rate during therapy phase (15%) than baseline phase (5%), visual inspection revealed overlapping points between the baseline and therapy phases, and mean accuracy during therapy phase did not exceed baseline trendline. The non-significant Tau-U test (Tau *U* = 0.75; *p* = 0.058) further indicated overlap between phases. In addition, there were non-significant WEST-ROC and WEST-Trend results, indicating the improvement is neither greater than the null ROC expected at baseline, nor greater than the baseline trend. MA4 did show a small effect size for acquisition (ES = 4.00). However, none of the methods provided large or significant support for acquisition following Fr-PCA for MA4.

BA1, who had moderate to severe aphasia and mild apraxia, showed strong evidence of acquisition following Fr-PCA. He demonstrated a greater mean accuracy rate during therapy phase (76%) than during baseline phase (7%), he did not show overlap between performance during therapy phase compared to baseline phase, and therapy improvements exceeded baseline trendline. This was confirmed by a significant Tau-*U* result (Tau *U* = 1.00; *p* = 0.008). When looking at the effect size, MA3 showed a large effect size for acquisition following Fr-PCA (ES = 15.30). Finally, both WEST-ROC and WEST-Trend yielded significant results. All four methods converge in supporting BA1’s strong acquisition following Fr-PCA.

BA2, who presented mild to moderate aphasia and mild to moderate apraxia, showed moderate evidence of acquisition following Fr-PCA. There was a greater mean accuracy rate during therapy phase (95%) than during baseline phase (50%), she did not show overlap between performance during therapy phase compared to baseline phase and showed therapy improvements that exceeded baseline trendline. This was confirmed by a significant Tau-*U* result (Tau *U* = 1.00; *p* = 0.008). When looking at the improvement during therapy phase, both WEST-ROC and WEST-Trend yielded significant results, indicating the ROC post-treatment was significantly greater than the null ROC and that improvement was above existing trends in the data. However, looking at the effect size, BA2 showed a moderate effect size for acquisition following Fr-PCA (ES = 9.00). Thus, only three methods give large or significant support of acquisition following Fr-PCA for BA2.

BA3, who had mild to moderate aphasia, showed moderate evidence of acquisition following Fr-PCA. He demonstrated a greater mean accuracy rate during the therapy phase (94%) than during the baseline phase (48%), did not show overlap between performance during therapy phase compared to baseline phase and showed therapy improvements that exceeded baseline trendline. This was confirmed by a significant Tau-*U* result (Tau *U* = 1.00; *p* = 0.008). However, looking at the improvement during therapy phase, although WEST-ROC yielded a significant result, WEST-Trend did not survive correction for multiple comparisons, indicating the improvement did not surpass existing trends in the data. Furthermore, looking at the effect size, BA3 showed only a moderate effect size for acquisition following Fr-PCA (ES = 8.95). Thus, only two of the four methods give large or significant support of acquisition following Fr-PCA for BA2.

BA4, who had moderate aphasia and mild apraxia, showed strong evidence of acquisition following Fr-PCA. He demonstrated a greater mean accuracy rate during the therapy phase (87%) than during the baseline phase (5%), he did not show overlap between performance during therapy phase compared to baseline phase and showed therapy improvements that exceeded baseline trendline. This was confirmed by a a significant Tau-*U* result (Tau *U* = 1.00; *p* = 0.008). When looking at the effect size, MA3 showed a large effect size for acquisition following Fr-PCA (ES = 14.00). Finally, both WEST-ROC and WEST-Trend yielded significant results. All four methods converge in supporting BA4’s strong acquisition following Fr-PCA.

In sum, for the mPWA group, one participant showed strong evidence of acquisition (MA3), one participant showed moderate acquisition (MA2), one participant showed weak evidence (MA1), and one participant showed no evidence (MA4). Figure 2 shows, for MA2, MA3 and MA4, some variability between therapy sessions in terms of performance. For the bPWA group, two participants showed strong evidence (BA1 and BA4), and two showed moderate evidence (BA2 and BA3) with more stable and rapid improvement rates.

#### Within-level generalization

**FIGURE 3:**
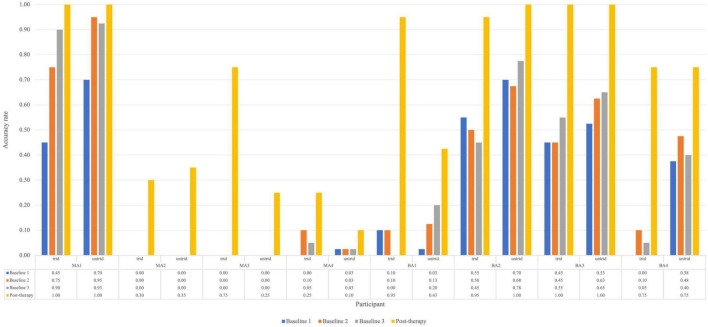
Accuracy rate for each participant for treated and untreated items at each picture-naming probe. Trtd, treated items (20) and untrtd, untreated items (40).

To examine within-level generalization, mixed-methods analyses were carried out. Two case series methods, namely analysis of effect size and WEST-ROC and WEST-Trend statistics, were employed to examine the improvement of untreated items. Figure 3 shows the response accuracy for each participant at assessment points for both treated and untreated items. The pre- and post-therapy scores on the TDQ60 – a francophone object-naming standardized task – were then compared to investigate the within-level generalization effect of Fr-PCA.

For untreated items, the analysis is based on baseline and post-therapy performance. When looking at Figure 2, all participants improved from the average baseline accuracy to post-therapy. Looking at the effect size of within-level generalization following Fr-PCA to untreated items, two participants show large effect sizes (MA2, ES = 26.06; MA3, ES = 18.61), four participants show small effect size of improvement on untreated (MA4, ES = 5.20; BA2, ES = 5.44; BA3, ES = 6.05; BA4, ES = 6.41), and two participants show non-significant effect size (MA1 and BA1). As for the WEST analysis, four participants show both significant WEST-ROC and WEST-Trend (MA2, MA3, BA3 and BA4). Additionally, one participant shows a significant WEST-ROC (BA3), and two show a significant WEST-Trend (MA1 and BA1).

Pre- and post-therapy *z*-scores, along with variation scores for the comprehensive language assessments, are included in Table 4. These scores were compared to assess within- and across-level generalization. A sign test^4^ was conducted to determine if Fr-PCA significantly affected participants’ performance variance pre-therapy to post-therapy on standardized language tests. When examining within-level generalization with regard to a standardized test, Fr-PCA led to statistically significant improvements for object naming (TDQ-60) within our participants (*p* = 0.016). Participants BA2 and BA3 performed within the normal range pre-therapy, and participant MA1 was at ceiling performance pre-therapy on this test. All other participants showed severe impairment (<–2 SD) and improved significantly following Fr-PCA. Please refer to the Supplementary material for a detailed description of the sign-test results in Table 4.

**TABLE 4:** Pre-therapy, post-therapy and variation of *z*-scores for all participants, exact sign test results testing the statistical difference between pre- and post-therapy for within-level generalization (TDQ60) and across-level generalization (DVL38, Verbal fluency, Repetition, oral comprehension, Cinderella story (MC)) and the Mann–Whitney *U* test results testing for statistical difference between groups’ variation scores.

		Standardized tests					Discourse
		TDQ60^†^	DVL38^†^	Verbal fluency^†^	Repetition^†^	Oral comprehension^†^	Cinderella story (MC)
MA1	Pre	1.032	0.046	–0.434	1.009	0.891	37
	Post	1.032	0.602	–0.273	1.009	0.891	39
	Variation	0.000	0.556	0.161	0.000	0.000	2
MA2	Pre	–14.148	–8.338	–3.660	–2.595	–7.525	12
	Post	–6.030	–6.629	–3.337	–0.793	–3.564	NR
	Variation	8.118	1.709	0.323	1.802	3.960	NR
MA3	Pre	–11.934	0.367	–3.836	0.699	–3.819	29
	Post	–5.292	0.451	–2.471	0.699	–2.079	66
	Variation	6.642	0.083	1.365	0.000	1.740	37
MA4	Pre	–25.851	–5.679	–3.982	–26.018	–6.535	4
	Post	–18.194	–3.020	–3.337	–23.315	–5.545	6
	Variation	7.658	2.659	0.645	2.703	0.990	2
BA1	Pre	–10.086	–0.695	–2.208	1.009	–0.099	25
	Post	–5.131	–0.046	–2.047	1.009	0.891	62
	Variation	4.955	0.649	0.161	0.000	0.990	37
BA2	Pre	–0.626	0.209	–1.724	–0.793	–0.594	27
	Post	0.725	0.209	–0.918	0.108	0.396	32
	Variation	1.351	0.000	0.806	0.901	0.990	5
BA3	Pre	–0.626	–1.066	–2.208	1.009	0.891	38
	Post	–0.176	0.324	–1.402	1.009	0.396	43
	Variation	0.450	1.390	0.806	0.000	–0.495	5
BA4	Pre	–6.932	0.417	–3.781	0.697	0.324	27
	Post	–4.230	0.510	–3.781	0.697	–0.099	45
	Variation	2.703	0.093	0.000	0.000	–0.423	18
Sign test standardized statistic		2.268	2.268	2.268	1.155	0.756	2.268
exact sig (two sided test) *p*-value		0.016	0.016	0.016	0.250	0.453	0.016
CI 95		[0.450–7.658]	[0.083–1.709]	[0.161–0.806]	[0.000–1.802]	[0.423–1.740]	[2–37]
Mean variation score for mPWA group (SD)		5.60 (3.79)	1.25 (1.16)	0.62 (0.53)	1.13 (1.35)	1.67 (1.68)	7.50 (20.60)
Mean variation score for bPWA group (SD)		2.36 (1.96)	0.53 (0.64)	0.44 (0.42)	0.23 (0.45)	0.27 (0.84)	16.25 (15.13)
Mann–Whitney *U* (stand. test statistic)		4.000 (–1.155)	5.000 (–0.866)	6.500 (–0.438)	5.000 (–0.992)	3.000 (–1.479)	12.500 (1.323)
exact sig. (two-sided) *p*-value		0.343	0.486	0.686	0.486	0.200	0.200
CI 95		[1.687–6.192]	[0.010–1.616]	[0.161–0.559]	[0.010–1.802]	[0.423–2.235]	[-16–3]

TDQ60, *Test de denomination de Québec* – object naming test; DVL38, *Dénomination de verbes lexicaux* verb naming test; *Tasks taken from the Montreal-Toulouse 86 Protocol – oral comprehension, repetition, verbal fluency*. † Scores presented are transformed *z*-scorez according to test administration booklet. MC, Main concept score calculated as per Richardson and Dalton (2016). NR, not reported. In green are the variation *z*-scores > 1 SD for normalized tests. SD, standard deviation. CI 95 for the sign test and the Mann–Whitney *U* test correspond to 95% confidence interval for the median value. mPWA were used as reference group for the calculation of the CI95 for the Mann–Whitney *U* test.

Clinically, a variation greater than +1 SD generally indicates a significant improvement. Following Fr-PCA, on the TDQ60, MA1 showed no variation as he was already at ceiling pre-therapy, and BA3 showed only slight improvement (+0.450). All other participants showed significant variation scores (MA2, MA3, MA4, BA1, BA2, BA4).

A Mann–Whitney *U* test was conducted to see if there was a difference between mPWA and bPWA in variations from pre-therapy to post-therapy on the TDQ60 performance (see Table 4). There was no statistically significant difference in the *z*-score variation between monolinguals and bilinguals.

In sum, the converging results of effect size and WEST statistics for the untreated item and the improvement on the TDQ60 suggest variable within-level generalization. MA2 and MA3 show strong evidence of within-level generalization, BA3 and BA4 show moderate evidence, MA4, BA1, and BA2 show weak evidence, whereas MA1 shows no evidence.

#### Across-level generalization

When measuring across-level generalization with standardized tests, Fr-PCA led to statistically significant improvements for verb naming (DVL-38), verbal fluency task, and narrative discourse informativeness, showing across-level generalization in all participants with PWA, except for MA1, who was within normal range for all standardized test and did not improve significantly for the main concept score. These results are displayed in Table 4.

Looking closer at the DVL38 results, in this sample, two participants showed severe impairments (MA2 and MA4) pre-therapy and made clinically significant gains (respectively +1.707 and +2.659). BA3, who showed impairments that did not quite reach the mild impairment criteria (*z*-score = –1.066), also made clinically significant gains. All other participants performed within the normal range and did not show clinically significant variations. Considering the verbal fluency task performance, among the seven participants with severe impairment (MA2, MA3, MA4, BA1, BA2, BA3, BA4), only one participant (MA3) made clinically significant improvements. Post-therapy, two participants (BA2 and BA3) made sufficient gains to no longer meet the criteria for a severe impairment. Finally, when looking at the main concept scores for the Cinderella discourse, all participants that completed the task at both assessments improved, though a wide range of gains was found (CI95 [2–37]).

To compare group differences (mPWA vs. bPWA), a Mann-Whitney U test was conducted using variation scores from the standardized test (see Table 4). None of the variation scores on standardized tests showed a statistically significant difference between monolinguals and bilinguals.

#### Transfer

Six PWA completed the Flanker task before and after therapy (two mPWA and four bPWA). Pre-therapy and post-therapy Flanker task outcome measures are included in Table 5. An exact sign test was conducted due to the asymmetrical distribution of differences. The sign test was conducted to determine if Fr-PCA had induced a statistically significant change in participants’ performance from pre-therapy to post-therapy on the Flanker task. For our sample of PWA, no statistically significant change in any cognitive control outcome measure (Flanker effect, error rate, and RT for both conditions) was found.

**TABLE 5:** Pre-therapy and post-therapy Flanker task outcome measure performances, exact sign test results testing the statistical difference between pre- and post-therapy, and Mann–Whitney *U* testing if groups significantly differ post-therapy.

		Flanker effect (ms)	Error rate		Response time (ms)	
			congruent	Incongruent	Congruent	Incongruent
MA1	Pre	293.74	0.03	0.19	739.37	1033.11
	Post	227.86	0.05	0.14	677.99	905.85
	Variation	–65.88	0.02	–0.05	–61.38	–127.26
MA2	Pre	NR	NR	NR	NR	NR
	Post	46.51	0.26	0.31	1321.46	1367.97
	Variation	NR	NR	NR	NR	NR
MA3	Pre	131.28	0.04	0.15	602.64	733.92
	Post	55.86	0.05	0.26	510.21	566.07
	Variation	–75.42	0.01	0.11	–92.43	–167.85
MA4	Pre	NR	NR	NR	NR	NR
	Post	193.50	0.28	0.61	1216.10	1409.60
	Variation	NR	NR	NR	NR	NR
BA1	Pre	95.74	0.00	0.01	697.65	793.38
	Post	158.26	0.00	0.01	738.09	896.36
	Variation	62.52	0.00	0.00	40.44	102.98
BA2	Pre	100.73	0.00	0.00	717.85	818.58
	Post	135.25	0.00	0.00	731.34	866.60
	Variation	34.52	0.00	0.00	13.49	48.02
BA3	Pre	142.64	0.01	0.01	911.29	1053.93
	Post	75.44	0.01	0.01	758.40	833.85
	Variation	–67.20	0.00	0.00	–152.89	–220.08
BA4	Pre	59.06	0.00	0.00	714.70	773.77
	Post	50.27	0.00	0.00	620.69	670.96
	Variation	–8.79	0.00	0.00	–94.01	–102.81
sign test standardized statistic		–0.408	0.707	0.000	–0.408	–0.408
exact sig (two sided test) *p*-value		0.688	0.500	1.000	0.688	0.688
CI95		[–75.42 – 62.52]	[0.00–0.02]	[–0.05 – 0.11]	[–152.89 – 40.44]	[–220.08 – 102.98]
Post-therapy mean (*SD*) mPWA		130.93 (93.22)	0.16 (0.13)	0.33 (0.20)	931.44 (397.83)	1062.37 (401.98)
Post-therapy mean (*SD*) bPWA		104.81 (50.40)	0.00 (0.00)	0.00 (0.00)	712.13 (62.04)	816.94 (100.62)
Mann–Whitney *U* (stand. test statistic)		7.000 (–0.289)	0.000 (–2.381)	0.000 (–2.337)	7.000 (–0.289)	4.000 (–1.155)
exact sig (two sided test) *p*-value		0.886	0.029	0.029	0.889	0.343
CI95		[–28.93 – 92.61]	[0.05–0.26]	[0.25–0.31]	[–80.41 – 563.06]	[9.49–534.12]

NR, not reported. In green are improvements (reduced error rate or reduced RT). SD, standard deviation. CI 95 for the sign test and the Mann–Whitney *U* test correspond to 95% confidence interval for the median value. mPWA were used as reference group for the calculation of the CI95 for the Mann–Whitney *U* test.

Looking at the Flanker effect and raw response times for both conditions, 4 of the 6 participants who completed the task at both assessments (MA1, MA3, BA3, BA4) improved in terms of interference suppression and speed. BA1 and BA2 showed increased response times post-therapy for both conditions and an increased Flanker effect. In terms of error rates, MA1 showed an improvement for the incongruent condition (–0.05 in error rate), whereas MA3 showed an important increase (+0.11 in error rate) at the post-therapy assessment. BA1, BA2, BA3, and BA4 did not show any variation; all demonstrated between 0.00 and 0.01 error rates in both conditions. It is noteworthy that two monolingual participants who were unable to perform the task before therapy were able to perform the task after, an improvement that statistical analysis cannot capture.

A Mann–Whitney *U* test was performed to determine if there is any difference between mPWA and bPWA in the post-therapy Flanker task performance (Table 5). There was no statistically significant difference between groups for the Flanker effect and raw RTs in the congruent and incongruent conditions. There was, however, a statistically significant difference between groups for error rates. In the congruent and incongruent conditions, mPWA (mean rank = 6.5) had a statistically significantly higher error rate than bPWA (mean rank = 2.5) (respectively, *U* = 0.000, *z* = –2.381, *p* = 0.029, *U* = 0.000, *z* = –2.337, *p* = 0.029).

Looking closer at the data, MA4 showed a lower Flanker effect than all bPWA, and MA3 showed a lower Flanker effect than BA1, BA2, and BA3, whereas MA1 and MA4 showed more significant Flanker effects than all bPWA. Regarding response times, MA2 and MA4 responded the slowest (RT > 1200 ms in both conditions). MA3 showed the fastest response times in all conditions, and MA1 showed faster response times than BA1, BA2, and BA3 for the congruent condition (at the cost of making more errors). In the incongruent condition, bPWA were faster than MA1, MA2, and MA4. In terms of error rates, in both conditions, mPWA showed higher error rates than bPWA (CI95 = [0.05–0.26] for the congruent condition and CI95 = [0.25–0.31] for the incongruent condition). MA3 was thus the fastest participant but maintained a higher error rate than bPWA.

### Impact of lesion location

A subset of ROIs damaged in at least two mPWA and two bPWA was identified to perform the statistical analysis. All participants have a single left hemisphere stroke; thus, the damaged ROIs are all in the left hemisphere. The Table in Supplementary Appendix B details the participants with damage to each ROI and the number of voxels damaged in each ROI per participant. The Mann–Whitney *U* test results are provided for all ROIs for which at least one participant per group shows damage. There was no significant difference between groups for lesion location and extent of damage (in voxel numbers).

The subset of ROIs damaged in at least two mPWA and two bPWA in our sample is composed of: the insular cortex, the frontal operculum cortex, the central opercular cortex, the frontal orbital cortex, the pars opercularis in the IFG, the MFG, the precentral gyrus, the postcentral gyrus, the temporal pole, Heschl’s gyrus (includes H1 and H2), the temporooccipital part of the MTG, the superior parietal lobule (SPL) and the planum polare. The subset covers ROIs throughout the frontotemporoparietal network known to be involved in language and cognitive control.

It is important to note that 4 mPWA showed damage to the posterior division of the SMG, but only one bPWA showed damage to this ROI. Similarly, three mPWA showed damage to the AnG, but no bPWA showed damage to this ROI. Three mPWA showed damage to the parietal operculum cortex, but only 1 bPWA showed damage to this ROI. However, these ROIs were not included in the correlation analysis because participants from only one group showed lesions in the areas.

#### Lesion location and behavioral performance pretherapy

A Spearman’s correlation was performed between the number of voxels within the ROIs covered with the lesion mask and the pretherapy language scores, and the Flanker task performance (for all participants; Table 6). There is a strong monotonic correlation between the pretherapy word naming score and the amount of damage in the MFG (TDQ; *r*_*s*_ = 0.740, *p* = 0.036) and the pars opercularis of the IFG (*r*_*s*_ = 0.740, *p* = 0.036). The pretherapy performance on the verbal fluency task correlated very strongly with the damage to MFG (*r*_*s*_ = 0.836, *p* = 0.010) and strongly with the amount of damage in the pars opercularis (*r*_*s*_ = 0.797, *p* = 0.018), the precentral gyrus (*r*_*s*_ = 0.712, *p* = 0.048), the temporal pole (*r*_*s*_ = 0.721, *p* = 0.044) and the anterior division of the STG (*r*_*s*_ = 0.768, *p* = 0.026).

**TABLE 6:** Significant correlations between damaged voxels within ROI and pre-therapy standardized language scores and cognitive control performance outcomes.

Language task	ROI	Spearman’s rho	*N*	*p*
TDQ	Middle Frontal Gyrus	0.740	8	0.036
	Inferior Frontal Gyrus, pars opercularis	0.740	8	0.036
Verbal fluency	Middle Frontal Gyrus	0.836	8	0.010
	Inferior Frontal Gyrus, pars opercularis	0.797		0.018
	Precentral Gyrus	0.712	8	0.048
	Temporal Pole	0.721	8	0.044
Flanker Effect	Inferior Frontal Gyrus, pars opercularis	0.820	6	0.046
	Temporal Pole	0.820	6	0.046
	Heschl’s gyrus (includes H1 and H2)	0.820	6	0.046
Flanker – congruent condition RT	Inferior Frontal Gyrus, pars opercularis	0.880	6	0.021
	Temporal Pole	0.880	6	0.021
	Postcentral Gyrus	0.941	6	0.005
	Frontal Operculum Cortex	0.912	6	0.011
	Central Opercular Cortex	0.928	6	0.008
	Parietal Operculum Cortex	0.845	6	0.034
	Planum Polare	0.928	6	0.008
	Heschl’s gyrus (includes H1 and H2)	0.880	6	0.021
Flanker – incongruent condition RT	Inferior Frontal Gyrus, pars opercularis	0.880	6	0.021
	Temporal Pole	0.880	6	0.021
	Postcentral Gyrus	0.941	6	0.005
	Central Opercular Cortex	0.841	6	0.036
	Parietal Operculum Cortex	0.845	6	0.034
	Planum Polare	0.801	6	0.036
	Heschl’s gyrus (includes H1 and H2)	0.880	6	0.021

The results presented do not survive correction for multiple comparisons.

When looking at the data, participants with the largest lesions in these ROIs performed well on the TDQ and the verbal fluency task pre-therapy, leading to a positive correlation. There were no significant correlations between the pretherapy scores on the DVL38, the oral comprehension task, the repetition task, and the narrative discourse task.

When considering the cognitive control task outcome measures, a strong positive correlation appears between the Flanker Effect and the damage to the pars opercularis (*r*_*s*_ = 0.820, *p* = 0.046), the temporal pole (*r*_*s*_ = 0.820, *p* = 0.046) and Heschl’s Gyrus (*r*_*s*_ = 0.820, *p* = 0.046) indicating difficulty in interference suppression with larger lesion size in these ROIs. The results presented do not survive correction for multiple comparisons.

#### Effect of lesion location on therapy outcome

A Spearman’s rank-order correlation was run to assess the relationship between the extent of the damage to ROIs and the variation of scores on linguistic tasks for all PWA (see Table 7). The variation score of the treated items correlated negatively with damage to the precentral gyrus (*r*_*s*_ = –0.878, *p* = 0.004), the central opercular gyrus (*r*_*s*_ = –0.826, *p* = 0.011), the MFG (*r*_*s*_ = –0.761, *p* = 0.028), the postcentral gyrus (*r*_*s*_ = –0.756, *p* = 0.030) and the SPL (*r*_*s*_ = –0.781, *p* = 0.022). The variation score of the untreated items correlated negatively to damage in the Heschl’s gyrus (*r*_*s*_ = –0.919, *p* = 0.001) and the postcentral gyrus (*r*_*s*_ = –0.761, *p* = 0.028). The change in the narrative discourse task is strongly correlated negatively to the damage in the insular cortex (*r*_*s*_ = –0.744, *p* = 0.034). These results showing negative correlations suggest that larger lesion size is related to less therapy-induced improvement in language performance. Finally, a strong positive correlation is found between the variation score in the repetition task and the damage in the SPL (*r*_*s*_ = 0.755, *p* = 0.030). However, this correlation is driven by participants without damage to the SPL performed at ceiling pretherapy and thus, did not show any variation. The results presented do not survive correction for multiple comparisons.

**TABLE 7:** Significant correlations between damaged voxels within ROI and variation scores.

Variation score	ROI	Spearman’s rho	*N*	*p*
Treated items	Middle Frontal Gyrus	–0.761	8	0.028
	Precentral Gyrus	–0.878	8	0.004
	Postcentral Gyrus	–0.756	8	0.030
	Superior Parietal Lobule	–0.781	8	0.022
	Central Opercular Cortex	–0.826	8	0.011
Untreated items	Postcentral Gyrus	–0.761	8	0.028
	Heschl’s gyrus (includes H1 and H2)	–0.919	8	0.001
Repetition	Superior Parietal Lobule	0.755	8	0.030
Cinderella	Insular Cortex	–0.744	8	0.034

The results presented do not survive correction for multiple comparisons.

### Role of right hemisphere

A Mann–Whitney *U* test was run with the cortical thickness generated by FreeSurfer to determine if there were differences between mPWA and bPWA. The ROIs with significant cortical thickness differences are presented in Table 8. For all ROIs that were significantly different between groups, the bPWAs have a significantly larger cortical thickness than mPWA, namely for the RH medial orbitofrontal gyrus, pars opercularis, precentral gyrus, rostral middle frontal gyrus, and frontal pole.

**TABLE 8:** Right hemisphere cortical thickness with significant group difference between monolingual and bilingual participants with aphasia.

	Medial orbitofrontal gyrus	Pars opercularis	Precentral gyrus	Rostral middle frontal gyrus	Frontal pole
MA1	2.173	2.336	2.257	1.865	2.135
MA2	2.199	2.037	2.236	2.06	2.087
MA3	2.117	2.365	2.262	2.042	2.228
MA4	2.255	2.162	2.185	2.025	2.217
BA1	2.457	2.424	2.433	2.202	2.537
BA2	2.256	2.393	2.367	2.237	2.400
BA3	2.295	2.366	2.264	2.067	2.422
BA4	2.429	2.678	2.699	2.34	2.549
Mean (*SD*) mPWA	2.186 (0.057)	2.225 (0.154)	2.235 (0.035)	1.998 (0.090)	2.167 (0.067)
Mean (*SD*) bPWA	2.359 (0.099)	2.465 (0.144)	2.441 (0.186)	2.211 (0.113)	2.477 (0.077)
Mann–Whitney *U* (stand. test statistic)	0.000 (–2.309)	0.000 (–2.309)	0.000 (–2.309)	0.000 (–2.309)	0.000 (–2.309)
exact sig. (two-sided) *p*-value	0.029	0.029	0.029	0.029	0.029
CI95	[–0.256 — –0.096]	[–0.342 — –0.059]	[–0.248 — –0.105]	[–0.298 — –0.160]	[–0.335 — –0.265]

SD, standard deviation. CI 95 for the Mann–Whitney U test corresponds to 95% confidence interval for the median difference value. mPWA were used as reference group for the calculation of the CI95.

#### Correlation with behavioral performances

A Spearman’s rank-order correlation assessed the relationship between the cortical thickness in the RH for ROIs that were significantly different between mPWA and bPWA and the linguistic and cognitive control tasks performances before therapy for all participants. Significant correlations were found only in the cognitive control task outcomes (see Table 9 for detailed statistical results). Namely, the Flanker effect was negatively correlated to the RH cortical thickness in the pars opercularis (*r*_*s*_ = –0.943, *p* = 0.005), the precentral gyrus (*r*_*s*_ = –0.943, *p* = 0.005), the rostral middle frontal gyrus (*r*_*s*_ = –0.886, *p* = 0.019) and the frontal pole (*r*_*s*_ = –0.829, *p* = 0.042), indicating less interference effect with larger cortical thickness for the undamaged ROIs in the RH. Also, there were negative correlations for these ROIs and the error rates in the congruent (medial orbitofrontal gyrus; *r*_*s*_ = –0.820, *p* = 0.046, pars opercularis; *r*_*s*_ = –0.880, *p* = 0.021, precentral gyrus; *r*_*s*_ = –0.880, *p* = 0.021 and rostral MFG; *r*_*s*_ = –0.880, *p* = 0.021) and the incongruent conditions (pars opercularis; *r*_*s*_ = –0.853, *p* = 0.031, precentral gyrus; *r*_*s*_ = –0.853, *p* = 0.03 and rostral MFG; *r*_*s*_ = –0.971, *p* = 0.001), indicating faster response time on cognitive control task is related to larger cortical thickness for these ROIs.

**TABLE 9:** Significant correlations between cortical thickness in right hemisphere ROIs and pre-therapy cognitive control task outcomes.

Language task	ROI	Spearman’s rho	*N*	*p*
Flanker Effect	Pars opercularis	–0.943	6	0.005
	Precentral gyrus	–0.943	6	0.005
	Rostral middle frontal gyrus	–0.886	6	0.019*
	Frontal pole	–0.829	6	0.042*
Flanker – congruent condition errors	Medial orbitofrontal gyrus	–0.820	6	0.046*
	Pars opercularis	–0.880	6	0.021*
	Precentral gyrus	–0.880	6	0.021*
	Rostral middle frontal gyrus	–0.880	6	0.021*
Flanker – incongruent condition error	Pars opercularis	–0.853	6	0.031*
	Precentral gyrus	–0.853	6	0.031*

*These results do not survive correction for multiple comparisons.

## Discussion

The purpose of this study was to examine the effects of intensive SLT with Fr-PCA in monolingual and bilingual participants with chronic aphasia while exploring its effects on both linguistic and cognitive performance, by reference to left hemisphere damage, and potential RH contribution to recovery. Eight PWA participated in this study, four French mPWA and four French-English bPWA. Neither bPWA reported nor demonstrated atypical language switching or mixing behavior (based on a spoken discourse performed in both languages) and showed better recovery of their L1, therefore qualifying for the French therapy program. All participants were compliant with therapy. To our knowledge, this is the first study investigating the effect of Fr-PCA across monolingual and bilingual PWA.

This study provides three main findings. First, Fr-PCA positively affected *acquisition* as demonstrated by the case series (improved accuracy in naming treated items), more so in bPWAs. Participants also showed *within-level generalization* (improved accuracy in naming untreated items), *across-level generalization* (improved performance in other linguistic tasks), and *transfer* (improvement on outcome measures from the Flanker task). Secondly, performance on linguistic and cognitive tasks before Fr-PCA was related to the location of left hemisphere damage, and so was therapy gain. Lastly, several frontal regions in the RH, known for their role in cognitive control, showed increased cortical thickness in bPWA compared to mPWA, and this difference was related to pre-therapy cognitive performance. The set of findings is discussed by reference to the impact of bilingualism on aphasia recovery and, more precisely, in relation to the question of Fr-PCA efficacy, the impact of lesion location and the role of the RH in recovery.

### Efficacy of French-Phonological Component Analysis

The first question investigated whether Fr-PCA would result in improvement for the *acquisition, within- and across-level generalization*, and *transfer* indices of treatment effects.

*Acquisition* In line with previous studies on the effect of PCA with mPWA (Leonard et al., 2008; Bose, 2013; Kristensson and Saldert, 2018; Marcotte et al., 2018), the results in this study show naming accuracy improvement for treated items following Fr-PCA, and extend these results to bPWA. Moreover, in this sample, bPWA show greater acquisition as measured by increased naming accuracy rate as compared to mPWA (bPWA: two participants show strong evidence, two participants show moderate evidence; mPWA: one participant shows strong evidence, one participant shows moderate evidence, one participant shows weak evidence, and one participant shows no evidence). More gain in acquisition for the bPWA finds support from a recent study showing the positive impact of bilingualism on phonological input processing using the oddball phonological paradigm measuring mismatch negativity (MMN; De Letter et al., 2021). De Letter et al. (2021) found a decrease in the latency of MMN in bPWA compared to monolingual peers suggesting that bPWA rely on their higher-order cognitive control network (which overlaps with the linguistic network; Abutalebi and Green, 2016) to restore their naming performance. In contrast, mPWA can only address the intact portions of the linguistic network, resulting in increased MMN latency and less recruitment of neurons underlying linguistic networks. Interestingly, strong evidence for improvement in our participants occurred in three participants (BA1, BA4, MA3) who had a smaller pre-therapy Flanker effect than other participants. The participant who showed weak/no evidence of improvement in acquisition either could not perform the flanker task (MA4) or had a substantial Flanker effect (MA1; 294 ms). These results suggest that cognitive control ability assessed by Flanker task performance may play a role in the treatment gain in acquisition. However, these findings contrast with Simic et al. (2020), who did not find any impact of cognitive control in immediate therapy gains but for generalization. On the other hand, MA2, who also could not complete the Flanker task pre-therapy, showed moderate evidence of acquisition following Fr-PCA. Thus, cognitive control abilities most likely interact with other abilities, allowing PWA to fully benefit from intensive SLT.

#### Within-level generalization

This study is also in line with previous studies showing improvement in naming accuracy rate for untreated items (Leonard et al., 2008) and extends these results to bPWA, confirming the hypothesis that Fr-PCA would lead to improvements in terms of within-level generalization. Concerning within-level generalization, strong evidence was seen for two of the mPWA (MA2, MA3), whereas bPWA showed moderate (BA2, BA4) to weak evidence (BA1, BA3) of improvement, based on the case-series analysis. However, a visual inspection of Figure 3 substantiates bPWA’s post-therapy within-level generalization gains; only they could name untreated items to a certain level at the baseline phase, lessening the results. In contrast, mPWA were unable to name untreated items at the baseline phase, except for MA1, resulting in more robust evidence of within-level generalization in case-series analysis. The strong evidence seen for mPWA is because they went from no naming performance to some naming performance for the untreated items. Indeed, bPWA exhibit more stable improvement or less variability for within-level generalization. This study observes within-level generalization beyond previous studies on PCA, with 7 out of 8 participants showing some evidence (Leonard et al., 2008). Our sample was similar to previous studies, and the protocol was identical, aside from the language used for therapy (first PCA study in French) and the impact of bilingualism. The multiple analysis methods in the case-series approach allowed a more detailed look at the within-level generalization. Most likely that using mean improvement in group analysis as a measure of improvement does not offer the sensitivity required to understand and discuss the trajectory of change in performance for within-level generalization following therapy targeting anomia.

#### Across-level generalization

The current study also finds evidence of across-level generalization following the Fr-PCA protocol. We find improvement on standardized language assessment for verb naming and verbal fluency. These findings suggest that naming improvements following Fr-PCA are not item specific, i.e., limited to words treated in therapy (Hickin et al., 2002), but extend to untreated word categories and language domains such as verbs and verbal fluency. This supports previous evidence that PCA facilitates picture naming through spreading activation within the phonological system (Goldrick and Rapp, 2002; Nickels, 2002), similar to the generalization process suggested following semantic-feature analysis (Boyle, 2004). Furthermore, phonological cues have been shown to improve naming abilities for most PWA compared to semantic and controlled cues (Meteyard and Bose, 2018).

Further looking at the effects of Fr-PCA on narrative discourse informativeness, main concept scores improved for all participants. Connected speech is considered the gold standard for aphasia rehabilitation and translation of therapy gains into everyday life improvement. It is one of the essential therapy outcomes for PWA (Kagan et al., 2008) and facilitates social participation (Carragher et al., 2012; Dalton and Richardson, 2015). Nevertheless, few anomia-therapy-efficacy studies measure improvement in connected speech (Peach and Reuter, 2009; Kristensson and Saldert, 2018). In the present study, bPWA showed generally higher main concept variation scores (+16.25, *SD* = 15.13) than monolinguals (+7.50, *SD* = 20.60), although this did not reach statistical significance. The bilingual group’s strengthened cognitive control mechanism could explain the larger across-level generalization seen in bPWA. Helm-Estrabrook and Ratner (2000) have suggested that cognitive control deficits lead to failure to generalize therapy gains into everyday communication through discourse. Furthermore, Penn et al. (2010) provide evidence for bPWA having better conversation strategies – good topic management, repair, and flexibility – and better cognitive control performance when compared to their monolingual peers. Thus, the current study supports across-level generalization in connected speech functions such as verbal fluency and discourse. For across-level generalization (verb naming, verbal fluency, and narrative discourse), no specific group difference was evident when bPWA performance was compared to mPWA, indicating insufficient evidence to conclude group-level differences in across-level generalization. A trend was, however, registered in the narrative discourse informativeness, advantaging bPWA.

#### Transfer

The hypothesis concerning transfer effects following Fr-PCA is partially confirmed. Although the statistical comparison between pre- and post-therapy was inconclusive, potentially due to the limited number of participants having completed the Flanker task at both pre- and post-therapy assessment sessions, we do find four participants reducing the Flanker effect (MA1, MA3, BA3, BA4), indicating less interference from distractors after intensive SLT. Two additional participants (MA2 and MA4) who could not perform the task during the pre-therapy assessment were able to perform the task after intensive SLT suggesting a therapy-induced change in cognitive control task performance. Two bPWA (BA1, BA2) demonstrated higher Flanker effects post-therapy (+62.5 ms, +34.52 ms), indicating more interference from distractors. At the post-therapy assessment, these participants verbalized specially focussing on gaining more accuracy in their performance, probably leading to a speed-accuracy trade-off. Although we do not observe any accuracy variation for these participants (both participants displayed a 0.00 error rate for the congruent condition and 0.01 for the incongruent condition), their Flanker effects were in typical ranges based on difference scores provided in previous literature (Calabria et al., 2019). Interestingly, upon comparing the post-therapy accuracy scores, bPWA did show significantly better performances than mPWA, indicating better cognitive performance on the task, which is in line with previous studies (Mooijman et al., 2021). Thus, the evidence supporting the transfer of gains to cognitive task performance following Fr-PCA therapy is based on the fact that all participants could successfully perform the task post-therapy compared to pre-therapy and either performed at an expected level or showed some level of improvement, either in terms of speed (MA1, MA3, BA3, BA4), accuracy (MA1) or reduced interference (MA1, MA3, BA3, BA4).

When looking at trends in the data, there is much more variability within the mPWA group than in the bPWA in relation to initial impairments and gains made following Fr-PCA. The interaction between the performance on the Flanker task and the linguistic abilities also seems more present in the bPWA group. However, it is difficult to conclude that Fr-PCA is related to transfer in cognitive performance. Indeed, bPWA’s better performance than their monolingual peers at the Flanker task in this study (reported in Table 5) replicates results from previous literature (Dash and Kar, 2014; Alladi et al., 2016; Paplikar et al., 2018; Dekhtyar et al., 2020; Lahiri et al., 2020; Penaloza et al., 2020; Mooijman et al., 2021). Therefore, it may also be possible that inhibition – a subcomponent of cognitive control examined by the Flanker task performance – may be a necessary pre-requisite for therapy gains and generalization (Yeung and Law, 2010), a question which needs to be addressed in future studies.

### Impact of lesion location

The hypothesis concerning the effect of lesion location on cognitive and linguistic profiles was partially confirmed. Contrary to previous findings (Sims et al., 2016; Daria et al., 2019), the evidence shows that larger lesions in the left IFG (pars opercularis) and MFG corresponded to higher pre-therapy picture-naming performance; three out of the four participants with lesions in these ROIs perform within the normal range for the TDQ60 (MA1, BA2, BA3; MA2 shows severe impairments on the TDQ60). In contrast, participants with no lesion in these same ROIs showed extensive picture naming difficulties (MA3, MA4, BA1, BA4). The nature of this interaction between lesion size in the left IFG and MFG and naming performance remains unclear. Previous findings have shown that vascular topography of stroke lesions and the corresponding impact on different behavioral performances may share variance with the lesion size and location (Sperber et al., 2020). However, vascular topography of lesions and corresponding lesion-symptom mapping is only possible with a larger sample.

In line with previous studies (Cole and Schneider, 2007; Abutalebi et al., 2008; Fridriksson, 2010), lesion location affects recovery – smaller lesion size in specific language and cognitive control networks were related to greater therapy outcomes for acquisition, within-, and across-level generalization (see Table 6). Lesions in the left postcentral gyrus are associated with phonological errors; the left postcentral gyrus plays a role in online somatosensory and auditory monitoring of articulation (Schwartz et al., 2012; Mirman et al., 2019), whereas phonological processing impairments involve lesions in Heschl’s gyrus (Ripamonti et al., 2018). Our results show that larger lesions in the left postcentral gyrus (MA1, MA2, MA4, BA2, BA3) and Heschl’s gyrus (MA1, MA4, BA2, BA3) led to less improvement in treated and untreated items, possibly indicating a lack of internalization of the strategy taught through Fr-PCA in the current study. The correlations in this study were not driven by any difference in groups in terms of the extent of damage within each ROI.

However, it is difficult to conclude that the trend of therapy-induced recovery differences between groups was not influenced by lesion location. Indeed, the mPWA in this sample showed more damage to the AnG (MA2, MA3, and MA4) and the SMG (MA1, MA2, MA3, MA4). None of the bPWA show damage to the AnG, and only BA3 shows damage to the SMG. Within the current cohort, MA2, MA3, and MA4 showed damage to the AnG, previously associated with less recovery (Fridriksson, 2010); it is relevant to imagine a relationship between the damage to the AnG and deficits in cognitive control. The AnG, part of the wider lateral parietal cortex, is involved in semantic memory and is associated with recollection in episodic memory (vs. familiarity) (Humphreys et al., 2021). It would have been interesting to see if a bilingualism-related cognitive control advantage would have continued with AnG damage. However, this was not possible within the sample at hand as none of the bPWA show damage to this ROI.

Further, another core region in the language network, the posterior division of the SMG (Blumstein and Baum, 2016), shows more damage in the mPWA group than in the bPWA group. The SMG has been previously linked to error detection and correction and, importantly, phonological error production (Blumstein and Baum, 2016). In the sample, the participant with the largest damage to the SMG (MA4) is the participant with the most severe naming impairment, however, this trend does not apply to the other participants.

Participants with larger lesions in central ROIs in the cognitive control network (Cole and Schneider, 2007; Abutalebi et al., 2008) did exhibit more interference effect and slower RTs on the Flanker task. It is known that the cognitive control network is vulnerable to lesions related to anterior aphasia (Keil and Kaszniak, 2002), with various studies showing deficits in cognitive control mechanisms in aphasia (Purdy, 2002; Dash et al., 2017). Impairments in the cognitive control mechanism are associated with poorer therapy outcomes, less generalization in therapy, and reduced functional communication skills (Fridriksson, 2010; Lambon Ralph et al., 2010; Simic et al., 2019). This is consistent with our findings, showing poorer therapy outcomes with lesions in the cognitive control network. However, with the current sample size, these findings can only be considered a trend and will need replication with larger study populations.

Finally, the lesion location data in this study also supports the relationship between cognitive control skills and discourse improvement, irrespective of the language group. We observe that more insular cortex damage (MA1, MA2, MA4, BA2, BA3, BA4) – part of the cognitive control network (Cole and Schneider, 2007) – triggers less improvement in narrative discourse performance. The insular cortex is also related to receptive language, expressive language, and language production (Oh et al., 2014). There was, however, no difference in the amount of damage to the insular cortex between groups. In sum, better therapy outcomes seen in bPWA compared to mPWA do not seem to be related to the amount of tissue damaged or location of the lesion. However, damage to the insular cortex is related to the discourse informativeness outcome across groups. This may indicate the therapy-specific impact of bilingualism-enhanced abilities, considering PCA treatment protocol relies heavily on cognitive control skills (Villard and Kiran, 2018). As developed by Leonard et al. (2008), the basic premise of PCA banks on cognitive control mechanisms as PCA requires actively generating or choosing between a succession of phonologically related cues to enhance the activation of phonological representations (Goldrick and Rapp, 2002) and ultimately improving word retrieval by internalizing the strategy. The succession of different cues for the same stimuli is particularly demanding in terms of inhibition – a subcomponent of cognitive control. bPWA may be more skilled in dealing with this constant demand.

### Role of right hemisphere

The third question looked at whether cortical thickness in the RH differed between bPWA and mPWA, given the role of RH in post-stroke language processing (or recovery) and bilingual experience-driven structural changes in the RH (Marin-Marin et al., 2022). Findings corroborated the prediction of differences in cortical thickness, where bPWA showed greater cortical thickness than mPWA in the medial orbitofrontal gyrus, the pars opercularis, the precentral gyrus, the rostral middle frontal gyrus and the frontal pole, areas involved in language and control network (Abutalebi and Green, 2016). Interestingly, with increased cortical thickness in the ROIs mentioned above, there is a decrease in interference effect and errors in flanker task performance, a trend indicating better cognitive control. In line with Gainotti (2015), structural differences such as the increased cortical thickness in the undamaged RH found in the bPWA group in this sample may indicate structural reserve in the bilingual group. The cortical thickness for pars opercularis and precentral gyrus (bPWA > mPWA) correlated negatively with the Flanker task outcomes (Flanker effect and error rates in both conditions), suggesting that cognitive performance is related to the undamaged RH.

So, improvements in the therapy outcomes in the bPWA may be related to the structural differences in the critical areas in the RH related to cognitive control that drive more benefits from intensive SLT like Fr-PCA therapy. In line with Lahiri et al. (2020), the current study found a trend in which bPWA showed better therapy-induced improvement on picture-naming probes compared to mPWA, implying a bilingual advantage. Similarly, the better accuracy on the Flanker task in bilinguals compared to monolinguals supports a bilingual advantage. Considering the literature on the bilingual advantage leading to more preserved cognitive control in bPWA, it is reasonable to suggest that the bilingual group benefits more from a cognitively demanding therapy such as Fr-PCA. Intact cognitive control mechanisms may not only be an indicator for successful PCA therapy outcomes; PCA therapy, in turn, can potentially enhance the cognitive control mechanisms and be beneficial for all participants.

### General discussion

All participants benefitted from Fr-PCA, either in the form of acquisition, generalization, or transfer, and demonstrated a variety of recovery profiles. The differences in severity of aphasia and variability in baseline performance of the participants could explain individual differences in the current study. Previous studies have reported that persons with more severe aphasia make less gains following therapy (Leonard et al., 2008; Kristensson and Saldert, 2018). Interestingly, participants with various impairment severity in the current study showed benefits from Fr-PCA. In seven participants out of eight showing some evidence of acquisition, difficulties ranged from mild to moderate (MA1), moderate (BA2, BA3, BA4), and moderate to severe (MA2, MA3, BA1). In line with a previous study by Kristensson and Saldert (2018), the participant with the most severe deficits (MA4) benefitted less than others from Fr-PCA by reference to the primary outcome measure (acquisition, improvement on treated items) but made important improvements on standardized tests and improved sufficiently in cognitive control to be able to complete the Flanker task post-therapy. It is possible that participants with severe aphasia might require longer stimulation protocols (Doogan et al., 2018) to achieve significant therapy gain. In the case of MA4, as tolerance throughout therapies increased, the participant accomplished more items in each therapy session. Similar to these findings, previous studies on PCA reported recovery in different aphasia profiles, usually concluding that individuals with severe aphasia benefit less (Leonard et al., 2008; Bose, 2013; Kristensson and Saldert, 2018; Marcotte et al., 2018). Variability in baseline performances of the participants may also predict improvements. Hence, Duncan et al. (2016) suggested that the pre-therapy variability in naming performance predicts a higher response to therapy. Accordingly, less intra-individual variability in naming performance pre-therapy could indicate a (near) maximal recovery (Duncan et al., 2016) and thus less benefits.

Interestingly, damage in the cognitive control and language network regions was related to greater pre-therapy interference from distractors (as indicated by higher Flanker effect) and slower RTs on the Flanker task pre-therapy, along with less improvement in the treated items (Fridriksson, 2010). This trend could, in line with previous results, strengthen the claim that reduced cognitive control is linked to less benefit following ortho-phonological therapy (Yeung and Law, 2010). Moreover, higher cortical thickness in bilinguals’ right homologous areas represents a structural difference associated with bilingualism (Olulade et al., 2016). The literature suggests that practicing two languages may increase neural reserve (Li et al., 2014; Abutalebi et al., 2015). The results of this work suggest that, in the event of stroke-induced damage to key cognitive control processing areas, bilingual adults with aphasia may perform better than their monolingual peers by mobilizing this neural reserve during therapy and achieve better recovery. It is possible that although our participants were similar in many ways (no significant difference in socio-demographic factors and pre-therapy measures of linguistic abilities, see Supplementary Appendix A), bPWA were more prepared for intensive SLT because of their better cognitive performance (measured by the Flanker task) and increased cortical thickness in the RH than monolinguals resulting in better treatment gains following Fr-PCA; namely in acquisition and discourse informativeness.

### Limitations

The number of participants in this study is higher than in previous works studying therapy-induced improvements following PCA. Still, a larger sample is required to argue better the role of bilingualism in following Fr-PCA-induced aphasia recovery, especially to corroborate the interpretations made from the neuroimaging data. The findings of this study should be interpreted cautiously, especially for the analyses based on subgroups of the total participants. These results should be considered a trend that can aid researchers in establishing sound hypotheses for future research with larger sample sizes.

Considerable heterogeneity is found in the participants with aphasia in the current study that limit the strength of the claims that can be made, even if this variability is well documented in the literature (Kiran et al., 2013). The variability in our sample could also explain why this study did not replicate Paplikar et al. (2018)’s finding that bilinguals have less severe aphasia. Indeed, MA1 did not perform like the other monolingual participants, showing less severe aphasia than the other bilingual participants. Also, as can be observed in Table 2, our bilingual sample is composed of late sequential bilinguals, usually with a professional goal of using a second language as motivation. They are now retired and use little English (L2) daily. This particular bilingual sample (in terms of characteristics) could also explain why we did not find correlations between the cognitive and linguistic profiles. Our findings are of new interest in the bilingual aphasia recovery literature, but these results cannot be generalized to the continuum of bilingualism.

### Future directions

Future research could explore the effect of bilingualism on cognitive control and aphasia recovery by focusing on inter-individual variability in different measures of bilingualism (Kuzmina et al., 2019; Penaloza et al., 2020), such as the effect of late acquisition of L2 and the proficiency.

Furthermore, a larger sample is required to understand the effect of different predictor variables – aphasia-related and lesion-related – in the context of bilingualism. Finally, considering the growing evidence on the network correlates of cognitive and neural advantages in bilinguals showing equivalent behavioral performances (Berroir et al., 2017), future studies on bilingual aphasia recovery should also include functional connectivity measures for an in-depth comprehension of how different pre-morbid network configurations might be at the source of better aphasia recovery in bilinguals with aphasia.

## Data availability statement

The raw data supporting the conclusions of this article will be made available by the authors, without undue reservation.

## Ethics statement

The studies involving human participants were reviewed and approved by CRIUGM’s Aging and Neuroimagery Research Ethics Committee. The patients/participants provided their written informed consent to participate in this study.

## Author contributions

MM-T: conceptualization, data curation, formal analysis, investigation, methodology, writing – original draft, and writing – review and editing. TD: conceptualization, formal analysis, supervision, and writing – review and editing. PB: formal analysis and methodology. AA: funding acquisition, resources, methodology, supervision, and writing – review and editing. All authors contributed to the article and approved the submitted version.

## Footnotes

1 Cognitive control will be the term used throughout this paper; it refers to cognitive mechanisms underlying the capacity to adapt and demonstrate flexibility regarding behaviors in a new condition and can be used synonymously with executive control and executive function (Dash & Ansaldo, 2017).

2
https://surfer.nmr.mgh.harvard.edu/

3 Or a Sign test when data did not respect assumptions.

4 A sign test was conducted due to the asymmetrical distribution of differences.
