# Supplementary material for: French Phonological Component Analysis and aphasia recovery: A bilingual perspective on behavioral and structural data
Source: Front Hum Neurosci. 2022 Sep 22;16:752121. doi: 10.3389/fnhum.2022.752121 (PMC9535680; doi:10.3389/fnhum.2022.752121)
Supplement: Supplementary file 1 [file Data_Sheet_1.docx]

Supplementary Material

# Supplementary Data

Pre-therapy, post-therapy and variation of raw scores for all participants and exact sign test results testing the statistical difference between pre- and post-therapy for within-level generalization (TDQ60) and across-level generalization (DVL38, Verbal fluency, Repetition, Oral Comprehension).

|  |  | Standardized tests | |  |  |  |
| --- | --- | --- | --- | --- | --- | --- |
|  |  | TDQ60 | DVL38 | Verbal fluency | Repetition | Oral Comprehension |
| MA1 | Pre | 60 | 103 | 22 | 30 | 47 |
|  | Post | 60 | 109 | 23 | 30 | 47 |
|  | Variation | 0 | 6 | 1 | 0 | 0 |
| MA2 | Pre | 18 | 15 | 2 | 26 | 30 |
|  | Post | 40 | 33 | 4 | 28 | 38 |
|  | Variation | 22 | 18 | 2 | 2 | 8 |
| MA3 | Pre | 24 | 99 | 0 | 30 | 37 |
|  | Post | 42 | 100 | 8 | 30 | 41 |
|  | Variation | 18 | 1 | 8 | 0 | 4 |
| MA4 | Pre | 1 | 43 | 0 | 0 | 32 |
|  | Post | 18 | 71 | 4 | 3 | 34 |
|  | Variation | 17 | 28 | 4 | 3 | 2 |
| BA1 | Pre | 36 | 95 | 11 | 30 | 45 |
|  | Post | 47 | 102 | 12 | 30 | 47 |
|  | Variation | 11 | 7 | 1 | 0 | 2 |
| BA2 | Pre | 57 | 105 | 14 | 28 | 44 |
|  | Post | 60 | 105 | 19 | 29 | 46 |
|  | Variation | 3 | 0 | 5 | 1 | 2 |
| BA3 | Pre | 57 | 91 | 11 | 30 | 47 |
|  | Post | 58 | 106 | 16 | 30 | 46 |
|  | Variation | 1 | 15 | 5 | 0 | -1 |
| BA4 | Pre | 43 | 107 | 7 | 30 | 46 |
|  | Post | 49 | 108 | 7 | 30 | 45 |
|  | Variation | 6 | 1 | 0 | 0 | -1 |
| TDQ60, *Test de denomination de Québec* – object naming test; DVL38, *Dénomination de verbes lexicaux ­*verb naming test; *Tasks taken from the Montreal-Toulouse 86 Protocol - oral comprehension, repetition, verbal fluency.* NR, not reported. | | | | | | |

# Effect of Fr-PCA – Details for sign-test

Within-level

Of the 8 PWA in the study, following Fr-PCA, 7 improved on the TDQ60 (MA1 showed a ceiling effect), a statistically significant median increase in the improvement (*Mdn* = 3.83) from pre-therapy (*Mdn* = -8.51) to post-therapy (*Mdn* = -4.68) with large effect size, *z* = 2.268, *p* = 0.016, r=0.80.

Across-level

On the DVL38, 7 participants improved, and BA2 remained the same, a statistically significant median increase in the improvement (*Mdn* = 0.60) from pre-therapy (*Mdn* = -0.32) to post-therapy (*Mdn* = 0.27) with large effect size, *z* = 2.268, *p* = 0.016, r=0.80. Seven participants improved on the verbal fluency task, and BA4 remained the same (although not a ceiling effect), a statistically significant median increase in the improvement (*Mdn* = 0.48) from pre-therapy (*Mdn* = -2.93) to post-therapy (*Mdn* = -2.25) with large effect size *z* = 2.268, *p* = 0.016, r=0.80. MA2, MA4 and, BA2 improved on the repetition task (the 5 other participants remained the same because of ceiling effect), no statistically significant median change (*Mdn* = 0.00) from pre-therapy (*Mdn* = 0.70) to post-therapy (*Mdn* = 0.70), *z* = 1.155, *p* = 0.250. For the oral comprehension task, 5 improved (MA2, MA3, MA4, BA1, and BA2), MA1 remained the same (ceiling effect), and BA3 and BA4 decreased slightly (less than 1 SD). There was no statistically significant median change (*Mdn* = 0.99) from pre-therapy (*Mdn* = -0.35) to post-therapy (*Mdn* = 0.15), *z* = 0.756, *p* = 0.453. Finally, for the variation in the main concept score for the Cinderella narrative discourse, 7 participants improved their score. MA2 participant did not perform the post-therapy and was excluded from the analysis. A statistically significant median improvement (*Mdn* = 5) from pre-therapy (*Mdn* = 27) to post-therapy (*Mdn* = 43) with a large size effect, *z* = 2.268, *p* = 0.016, r=0.86. When performing the statistical test for each group separately on all standardized tests, improvements fail to reach significant levels.
