# Supplementary material for: French Phonological Component Analysis and aphasia recovery: A bilingual perspective on behavioral and structural data
Source: Front Hum Neurosci. 2022 Sep 22;16:752121. doi: 10.3389/fnhum.2022.752121 (PMC9535680; doi:10.3389/fnhum.2022.752121)
Supplement: Supplementary file 2 [file Data_Sheet_2.docx]

Appendix A. Comparative statistics (Mann-Whitney U test) between groups for sociodemographic factors and pretherapy assessment scores.

|  |  | Mean rank | | U | Z-score | p-value |
| --- | --- | --- | --- | --- | --- | --- |
|  |  | mPWA | bPWA |  |  |  |
| Sociodemographic factors | Age | 5.00 | 4.00 | 6.000 | -0.577 | 0.686 |
|  | Years of education | 2.75 | 6.25 | 15.000 | 2.084 | 0.057 |
|  | Time post-stroke | 5.75 | 3.25 | 3.000 | -1.452 | 0.200 |
|  | Lesion size | 5.25 | 3.75 | 5.000 | -0.866 | 0.486 |
|  | MoCA |  |  | 8.000 |  | 0.629 |
| Pretherapy language scores | Picture-naming probe accuracy rate | 3.50 | 5.50 | 12.000 | 1.156 | 0.343 |
|  | TDQ60 | 3.50 | 5.50 | 12.000 | 1.162 | 0.343 |
|  | DVL38 | 3.75 | 5.25 | 11.000 | 0.866 | 0.486 |
|  | Oral comprehension | 3.38 | 5.63 | 12.500 | 1.307 | 0.200 |
|  | Repetition | 3.75 | 5.25 | 11.000 | 0.887 | 0.486 |
|  | Verbal fluency | 3.75 | 5.25 | 11.000 | 0.871 | 0.486 |
|  | Main concept score for the Cinderella story | 4.00 | 5.00 | 10.000 | 0.581 | 0.686 |

Appendix B:

Number of participants with damage to each ROIs, number of voxels damaged in each ROI per participant and Mann-Whitney statistical results testing for a difference between groups for the ROIs identified in the subset for correlation analysis (in bold).

|  | Total number of participants with damaged ROI | mPWA participants with damaged ROI | bPWA participants with damaged ROI | Number of lesioned voxels | | | | | | | | Mann-Whitney U | Z | Exact Sig. |
| --- | --- | --- | --- | --- | --- | --- | --- | --- | --- | --- | --- | --- | --- | --- |
|  |  |  |  | MA1 | MA2 | MA3 | MA4 | BA1 | BA2 | BA3 | BA4 |  |  |  |
| Frontal Pole | 3 | 2 | 1 | 21205 | 502 | 0 | 0 | 0 | 0 | 197 | 0 | 0.000 | -1.225 | 0.667 |
| **Insular Cortex** | **6** | **3** | **3** | **9890** | **8426** | **0** | **1010** | **0** | **993** | **6599** | **7623** | **2.000** | **-1.091** | **0.400** |
| Superior Frontal Gyrus | 2 | 1 | 1 | 3270 | 0 | 0 | 0 | 0 | 267 | 0 | 0 | **0.000** | **-1.000** | **1.000** |
| **Middle Frontal Gyrus** | **4** | **2** | **2** | **19353** | **2277** | **0** | **0** | **0** | **11672** | **3794** | **0** | **2.000** | **0.000** | **1.000** |
| Inferior Frontal Gyrus, pars triangularis | 3 | 2 | 1 | 5183 | 260 | 0 | 0 | 0 | 0 | 4199 | 0 | 1.000 | 0.000 | 1.000 |
| **Inferior Frontal Gyrus, pars opercularis** | **4** | **2** | **2** | **6131** | **2921** | **0** | **0** | **0** | **4642** | **5880** | **0** | **2.000** | **0.000** | **1.000** |
| **Precentral Gyrus** | **5** | **3** | **2** | **18290** | **5183** | **0** | **4088** | **0** | **11125** | **5385** | **0** | **2.000** | **-0.577** | **0.800** |
| **Temporal Pole** | **4** | **2** | **2** | **375** | **186** | **0** | **0** | **0** | **29** | **336** | **0** | **1.000** | **-0.775** | **0.667** |
| Superior Temporal Gyrus, anterior division | 2 | 1 | 1 | 121 | 0 | 0 | 0 | 0 | 29 | 0 | 0 | 0.000 | -1.000 | 1.000 |
| Superior Temporal Gyrus, posterior division | 2 | 1 | 1 | 0 | 0 | 0 | 30 | 75 | 0 | 0 | 0 | 0.000 | -1.000 | 1.000 |
| Middle Temporal Gyrus, posterior division | 1 | 0 | 1 | 0 | 0 | 0 | 0 | 1728 | 0 | 0 | 0 | N.C. | N.C. | N.C. |
| **Middle Temporal Gyrus, temporooccipital part** | **3** | **2** | **1** | **0** | **0** | **241** | **26** | **1512** | **0** | **0** | **0** | **0.000** | **-1.225** | **0.667** |
| Inferior Temporal Gyrus, posterior division | 1 | 0 | 1 | 0 | 0 | 0 | 0 | 874 | 0 | 0 | 0 | N.C. | N.C. | N.C. |
| Inferior Temporal Gyrus, temporooccipital part | 1 | 0 | 1 | 0 | 0 | 0 | 0 | 229 | 0 | 0 | 0 | N.C. | N.C. | N.C. |
| **Postcentral Gyrus** | **5** | **3** | **2** | **7637** | **643** | **0** | **21751** | **0** | **2854** | **11503** | **0** | **3.000** | **0.000** | **1.000** |
| **Superior Parietal Lobule** | **5** | **3** | **2** | **142** | **145** | **0** | **10995** | **0** | **1255** | **499** | **0** | **2.000** | **-0.577** | **0.800** |
| Supramarginal Gyrus, anterior division | 3 | 2 | 1 | 248 | 0 | 0 | 5760 | 0 | 0 | 5007 | 0 | 1.000 | 0.000 | 1.000 |
| Supramarginal Gyrus, posterior division | 5 | 4 | 1 | 21 | 309 | 147 | 6617 | 0 | 0 | 1569 | 0 | 1.000 | -0.707 | 0.800 |
| Angular Gyrus | 3 | 3 | 0 | 0 | 1677 | 303 | 5402 | 0 | 0 | 0 | 0 | N.C. | N.C. | N.C. |
| Lateral Occipital Cortex, superior division | 3 | 2 | 1 | 0 | 3790 | 0 | 11146 | 0 | 5884 | 0 | 0 | 1.000 | 0.000 | 1.000 |
| Lateral Occipital Cortex, inferior division | 0 | 0 | 0 | 0 | 0 | 0 | 0 | 0 | 0 | 0 | 0 | N.C. | N.C. | N.C. |
| Juxtapositional Lobule Cortex (formerly Supplementary Motor Cortex) | 1 | 1 | 0 | 1197 | 0 | 0 | 0 | 0 | 0 | 0 | 0 | N.C. | N.C. | N.C. |
| Paracingulate Gyrus | 0 | 0 | 0 | 0 | 0 | 0 | 0 | 0 | 0 | 0 | 0 | N.C. | N.C. | N.C. |
| Cingulate Gyrus, anterior division | 1 | 1 | 0 | 1143 | 0 | 0 | 0 | 0 | 0 | 0 | 0 | N.C. | N.C. | N.C. |
| Cingulate Gyrus, posterior division | 2 | 2 | 0 | 311 | 0 | 0 | 217 | 0 | 0 | 0 | 0 | N.C. | N.C. | N.C. |
| Precuneous Cortex | 1 | 1 | 0 | 0 | 0 | 0 | 2439 | 0 | 0 | 0 | 0 | N.C. | N.C. | N.C. |
| **Frontal Orbital Cortex** | **4** | **2** | **2** | **9175** | **1135** | **0** | **0** | **0** | **0** | **1620** | **717** | **1.000** | **-0.775** | **0.667** |
| Lingual Gyrus | 0 | 0 | 0 | 0 | 0 | 0 | 0 | 0 | 0 | 0 | 0 | N.C. | N.C. | N.C. |
| Occipital Fusiform Gyrus | 0 | 0 | 0 | 0 | 0 | 0 | 0 | 0 | 0 | 0 | 0 | N.C. | N.C. | N.C. |
| **Frontal Operculum Cortex** | **5** | **2** | **3** | **2819** | **2162** | **0** | **0** | **0** | **118** | **2819** | **647** | **1.500** | **-0.889** | **0.400** |
| **Central Opercular Cortex** | **6** | **3** | **3** | **7613** | **4818** | **0** | **2772** | **0** | **5904** | **6991** | **1312** | **4.000** | **-0.218** | **1.000** |
| Parietal Operculum Cortex | 4 | 3 | 1 | 1282 | 529 | 0 | 4437 | 0 | 0 | 4205 | 0 | 1.000 | -0.447 | 1.000 |
| **Planum Polare** | **5** | **2** | **3** | **752** | **205** | **0** | **0** | **0** | **191** | **280** | **78** | **1.000** | **-1.155** | **0.400** |
| **Heschl's Gyrus (includes H1 and H2)** | **4** | **2** | **2** | **317** | **0** | **0** | **1145** | **0** | **61** | **298** | **0** | **0.000** | **-1.549** | **0.333** |
| Planum Temporale | 3 | 2 | 1 | 72 | 0 | 0 | 2489 | 0 | 0 | 554 | 0 | 1.000 | 0.000 | 1.000 |
| Occipital Pole | 1 | 0 | 1 | 0 | 0 | 0 | 0 | 0 | 0 | 0 | 21 | N.C. | N.C. | N.C. |
| The subsets of ROIs used to conduct the correlation analysis are identified in bold. The are the ROIs for which at least 2 monolingual and 2 bilingual participants with aphasia have damaged voxels.  Mann-Whitney U test was performed for all possible ROIs, where at least one mPWA and one bPWA have damaged voxels. For the rest of the ROIs where N =0 for mPWA or BPWA within the lesion mask, the Mann-Whitney U test could not be computed (marked N.C. in the Table). | | | | | | | | | | | | | | |
